# Thrombin-Derived Peptides Potentiate the Activity of Gram-Positive-Specific Antibiotics against Gram-Negative Bacteria

**DOI:** 10.3390/molecules26071954

**Published:** 2021-03-30

**Authors:** Charlotte M. J. Wesseling, Thomas M. Wood, Cornelis J. Slingerland, Kristine Bertheussen, Samantha Lok, Nathaniel I. Martin

**Affiliations:** 1 Biological Chemistry Group, Institute of Biology Leiden, Leiden University, 2333 Leiden, The Netherlands; c.m.j.wesseling@biology.leidenuniv.nl (C.M.J.W.); t.m.wood@biology.leidenuniv.nl (T.M.W.); c.j.slingerland@biology.leidenuniv.nl (C.J.S.); k.bertheussen@lic.leidenuniv.nl (K.B.); samanthalok1@hotmail.nl (S.L.); 2 Department of Chemical Biology & Drug Discovery, Utrecht Institute for Pharmaceutical Sciences, Utrecht University, 3584 Utrecht, The Netherlands; 3 Bio-Organic Synthesis Group, Leiden Institute of Chemistry, Leiden University, 2333 Leiden, The Netherlands

**Keywords:** LPS-targeting peptides, outer membrane disruption, antibiotic synergy, checkerboard assays

## Abstract

The continued rise of antibiotic resistance threatens to undermine the utility of the world’s current antibiotic arsenal. This problem is particularly troubling when it comes to Gram-negative pathogens for which there are inherently fewer antibiotics available. To address this challenge, recent attention has been focused on finding compounds capable of disrupting the Gram-negative outer membrane as a means of potentiating otherwise Gram-positive-specific antibiotics. In this regard, agents capable of binding to the lipopolysaccharide (LPS) present in the Gram-negative outer membrane are of particular interest as synergists. Recently, thrombin-derived C-terminal peptides (TCPs) were reported to exhibit unique LPS-binding properties. We here describe investigations establishing the capacity of TCPs to act as synergists with the antibiotics erythromycin, rifampicin, novobiocin, and vancomycin against multiple Gram-negative strains including polymyxin-resistant clinical isolates. We further assessed the structural features most important for the observed synergy and characterized the outer membrane permeabilizing activity of the most potent synergists. Our investigations highlight the potential for such peptides in expanding the therapeutic range of antibiotics typically only used to treat Gram-positive infections.

## 1. Introduction

The rising tide of antibiotic resistance presents a clear threat to global health. This threat, coupled with the well-documented dearth of new antibiotics in the development pipeline, means that resistant pathogens are even more problematic [1]. Based on current trends, the World Health Organization (WHO) predicts that infections due to resistant bacteria will be the leading cause of death globally by 2050 [2]. The WHO recently published its updated “pathogen threat list” of which three drug-resistant Gram-negative species were assigned top priority: carbapenem-resistant *Acinetobacter baumannii*, *Pseudomonas aeruginosa*, and *Enterobacteriaceae* [3]. The emergence and proliferation of such resistant Gram-negative pathogens is concerning given the limited number of viable treatment options available [3]. 

**Figure 1:**
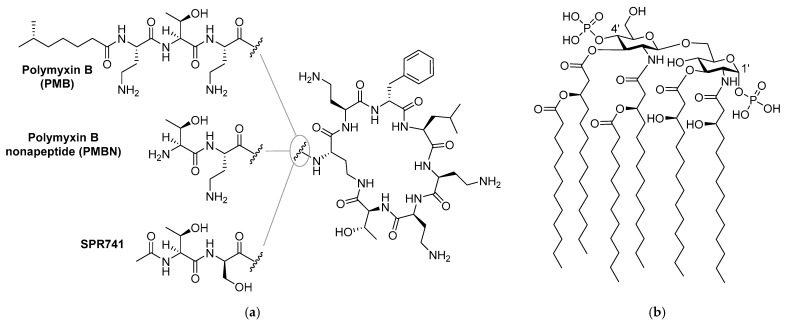
Structures of: (**a**) polymyxin B (PMB), polymyxin B nonapeptide (PMBN), and SPR741; (**b**) lipid A component of the Gram-negative lipopolysaccharide (LPS).

It is well established that compared to Gram-positive pathogens, Gram-negative bacteria are more difficult to kill with antibiotics due to the presence of an additional barrier: the outer membrane (OM) [4,5]. The OM protects Gram-negative bacteria from a large number of antibiotics that are used clinically to treat infections with Gram-positive bacteria [4]. Disruption of the OM has been widely investigated and in some cases proven to be an effective method to enable such antibiotics to function against Gram-negative bacteria [6,7,8,9,10]. In this regard, combinations of a membrane disruptor such as the well-studied polymyxin B nonapeptide (PMBN, Figure 1) along with macrolides or rifamycin-type antibiotics represent classic examples of such synergistic activity: the use of a combination leads to better results than a sum of each of the separate components [6,7,11,12,13,14]. Notably, the polymyxin derivative SPR741 (Figure 1), a selective OM disruptor developed by Spero Therapeutics, recently passed Phase I clinical trials [13,15,16].

Like its parent polymyxin B (Figure 1), SPR741 targets the bacterial lipopolysaccharide (LPS), a major component on the OM outer leaflet [4,14,17]. The core of LPS consists of Lipid A, a heavily lipidated disaccharide bearing phosphate groups at the 1’ and 4’ positions (Figure 1) [4]. Small cations such as Mg^2+^ and Ca^2+^ bridge the negative charges of the phosphate groups and in doing so contribute to the tight packing of LPS [4,6]. It is generally held that highly positively charged compounds such as PMBN bind the negatively charged LPS with high affinity and in doing so interfere with LPS packing, leading to OM permeabilization [7,18,19,20,21].

Compounds that bind to LPS with high affinity are also often referred to as endotoxin neutralizing compounds. Such compounds can demonstrate beneficial effects in reducing the inflammatory responses associated with systemic LPS exposure as in the case of sepsis [22,23,24,25]. In recent years, an increasing number of reports have appeared describing the synergistic effects of various positively charged small molecules and peptide-based compounds that interact with LPS [24,25,26,27,28,29,30,31,32,33].

Given the apparent link between LPS binding, OM permeabilization, and antibiotic potentiation, we set out to identify literature compounds described as having affinity for LPS that had not yet been evaluated for synergy with Gram-positive-specific antibiotics. This led us to the family of thrombin-derived C-terminal peptides (TCPs) reported by Schmidtchen and coworkers. In 2010, the Schmidtchen group first reported that peptide fragments from the C-terminus of thrombin, a key enzyme in the coagulation cascade, exhibit activity as host-defense peptides [34]. Subsequent structure activity studies by the same group identified peptide sequences with optimal antibacterial activity and recent NMR studies have elucidated the structural basis for their interaction with LPS [26,35,36].

In the present study we set out to assess the potential for TCPs to potentiate the anti-Gram-negative activity of otherwise inactive Gram-positive-specific antibiotics. To do so, synergy assays were first conducted using the TCPs described in the literature in combination with various antibiotics. Our initial studies revealed that, as we had hypothesized, the TCPs do indeed exhibit synergy. Building from these results we then prepared a number of new peptide analogues to assess the structural elements most important for synergy. Notably, we found that synergistic activity does not necessarily directly correlate with the inherent antibacterial activity of these peptides. We here report several new peptides inspired by thrombin-derived C-terminal peptides that display enhanced synergistic effects and reduced hemolytic activity.

## 2. Results

### 2.1. Synergy with Thrombin-Derived C-terminal Peptides

**Figure 2:**
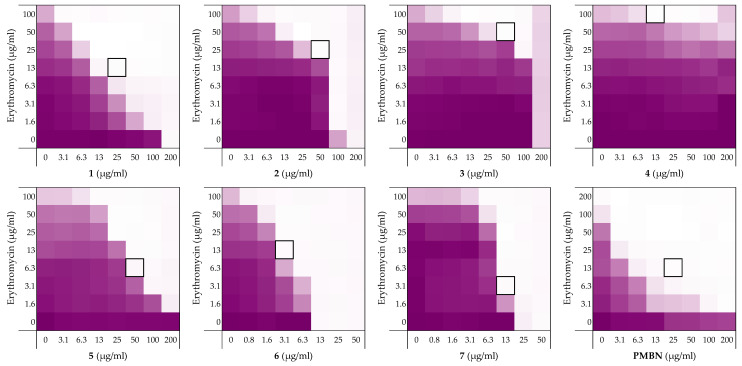
Checkerboard assays of the peptides **1**–**7** and PMBN in combination with erythromycin versus *E. coli* BW25113. In each case, the bounded box in the checkerboard assays indicates the combination of peptide and antibiotic resulting in the lowest FICi (see Table 1). The optical density at 600 nm (OD_600_) were measured using a plate reader and transformed to a gradient: purple represents growth, white represents no growth. An overview of all checkerboard assays with erythromycin can be found in the Supplementary data, Figure S1.

To begin, we selected four peptides previously described by the Schmidtchen group as LPS-binding [26]. The sequences of these peptides (**1**–**4**) are provided in Table 1 and range in length from 12 to 25 amino acids. Common to all four is the core sequence previously reported to be responsible for LPS binding [26]. The peptides were readily synthesized using solid-phase peptide synthesis (SPPS) and screened for synergistic activity using checkerboard assays. Synergy was initially assessed in combination with erythromycin (Figure 2, also see Supplementary data, Figure S1) and rifampicin (See Supplementary data, Figure S2 in Lysogeny Broth (LB) using *Escherichia coli* BW25113 as the indicator strain. Synergy is quantified by means of the fractional inhibitory concentration index (FICi) where an FICi of ≤0.5 is defined as synergistic and the lower the value, the more synergistic the combination [37]. 

Prior to assessing synergy, the MICs of the peptides themselves were measured revealing that they exhibit little-to-no inherent activity with MICs equal to, or above, the maximum 200 µg/mL concentration tested. The MICs of the companion antibiotics erythromycin and rifampicin were measured to be 100–200 µg/mL and 8 µg/mL, respectively (see Supplementary data, Tables S1 and S2). Using these parameters checkerboard assays were performed as illustrated in Figure 2. The results of the checkerboard assays performed with peptides **1**–**4** reveal clear differences in their synergistic potential. While the shortest peptide **1** exhibits potent synergy with erythromycin, the longer peptides **2**–**4** demonstrate comparatively little or no synergy (Table 1). In combination with rifampicin, peptides **1**–**4** all showed some synergistic activity with FICi’s ranging from 0.094 to 0.313, but with peptide **1** again displaying the most potent synergy (see Supplementary data, Table S2). These preliminary findings served to validate our hypothesis that LPS-binding peptides derived from thrombin have the capacity to synergize with Gram-positive specific antibiotics. All peptides were also screened for hemolytic activity which revealed a clear trend: while the shorter peptides **1** and **2** showed no appreciable hemolytic activity, the longer peptide **3** and **4** were highly hemolytic (Table 1). This hemolysis data, combined with the synergistic activity observed, led us to select peptide **1** for further investigation.

Building on these findings and with peptide **1** as our lead synergist, we next explored the impact of changes to the N- and C-termini of the peptide. To this end, peptides **5**–**7** were prepared to examine the impact of N-terminal acetylation and/or C-terminal amidation. N-terminal acetylation alone as in peptide **5** was found to have minimal effect on the inherent activity or synergistic potential of the peptide. By comparison, C-terminal amidation as in peptides **6** and **7** led to a significant increase in the inherent antibacterial activity with little impact on hemolytic activity. The reduced MIC values thus achieved, particularly notable for peptide **6**, provides a key advantage in that a lower concentration of peptide is required to achieve synergy: peptide **6** has an MSC of 3.125 µg/mL versus 25 µg/mL of its parent peptide **1** (Table 1). To assess whether peptide **6** employs an LPS mediated mechanism of action similar to peptide **1**, an LPS competition assay was also performed. Notably, the MIC of peptide **6** was found to increase from 12.5 µg/mL to 200 µg/mL in the presence of 1 mg/mL of LPS (See Supplementary data, Figure S4 and Table S4). This finding indicates that the antimicrobial activity of peptide **6** relies on LPS binding. Based on its enhanced activity and confirmed LPS-dependent mechanism, we next took peptide **6** forward for additional structure-activity studies by means of an alanine scan.

### 2.2. Alanine Scan of Peptide 6

**Figure 3:**
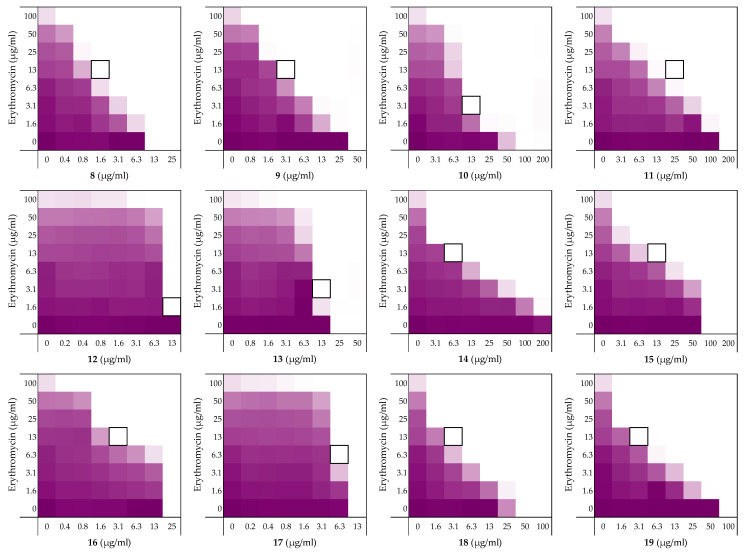
Checkerboard assays of the Ala-scan peptides **8**–**19** in combination with erythromycin versus *E. coli* BW25113. In each case the bounded box in the checkerboard assays indicates the combination of peptide and antibiotic resulting in the lowest FICi (see Table 2). OD_600_ values were measured using a plate reader and transformed to a gradient: purple represents growth, white represents no growth. An overview of all checkerboard assays with erythromycin can be found in the Supplementary data, Figure S1.

To assess the role of the individual amino acids in peptide **6** and their specific contribution to both the inherent activity and synergistic activity of the peptide, an alanine scan was performed. Like the parent peptide, peptides **8**–**19** (Table 2) were synthesized as the C-terminus amides using microwave-assisted automated SPPS. As summarized in Table 2, the MICs of the alanine scan peptides ranged from 12.5 µg/mL, as for peptide **6**, to above the maximum concentration tested of 200 µg/mL. After establishing the individual MICs for peptides **8**–**19**, checkerboard assays were performed as shown in Figure 3. The FICi values thus obtained clearly show that the alanine exchange introduced in peptides **12**, **13**, and **17** leads to a complete loss of synergistic activity. Notably, the common feature in these three peptides is the replacement of a lysine residue with alanine. Moreover, while no longer synergistic with erythromycin, these peptides still have a relatively low MIC of 25 µg/mL. Hemolysis data offers insight into this trend: the KΔA peptides **12**, **13**, and **17** are all hemolytic, which suggests a nonselective membrane disruption mode of action. By comparison, none of the other alanine scan peptides show appreciable hemolytic activity (Table 2).

Somewhat surprisingly, all of the other peptides prepared in the alanine scan study were found to exhibit more potent synergistic activity than peptide **6**. Notably, these peptides all exhibit synergy at concentrations lower than required for PMBN (see Table 1 and Table 2). In addition, an apparent trend emerges from the alanine scan data where decreased antimicrobial activity is inversely proportional to the synergistic activity of the peptides.

Among the non-hemolytic peptides generated in the alanine scan, only peptide **8** (V^1^ΔA) retains the same inherent antimicrobial activity as peptide **6** with an MIC of 12.5 µg/mL. It is, however, interesting to note that while replacement of other hydrophobic amino acids in peptide **6** with alanine as for **9**, **11**, **14**, **15**, **18**, and **19** did result in a decrease of antimicrobial activity, it also led to significant enhancement of synergistic activity (Table 2). Apparently, replacing the bulkier aromatic side-chains as in F^2^ΔA (**9**) and W^7^ΔA (**14**) is an especially favorable exchange when it comes to potentiating the activity of erythromycin. Notably, replacement of the C-terminal Ile residue as for I^12^ΔA (**19**) results in a strongly synergistic peptide, while replacing Ile in the center of the peptide as in I^8^ΔA (**15**) has a less profound effect. Moreover, while peptide **9** has the same FICi as PMBN, peptides **14** and **19** are even more potent synergists.

As mentioned above, the cationic side-chains of the Lys residues present in peptide **6** are required for synergy and also serve to limit hemolysis. By comparison, alanine replacement of the polar but neutral glutamine, as in Q^9^ΔA (**16**), appears to have little effect. Moreover, the R^3^ΔA substitution in peptide **10**: the only other case wherein a positively charged side-chain was replaced by alanine, did not trigger hemolytic activity and retained synergistic activity. We therefore decided to also take peptide **10** along with a broader screening of the most potent synergistic peptides **14** and **19**.

### 2.3. Exploring the Synergistic Range

A well-studied example of synergy is the potentiation of erythromycin and rifampicin against Gram-negative bacteria by PMBN. Clinically, erythromycin and rifampicin are generally only used to treat infections due to Gram-positive pathogens as both exhibit rather limited activity against Gram-negative strains [38,39,40]. Other Gram-positive specific antibiotics, such as novobiocin and vancomycin, have also been shown to be capable of killing Gram-negative pathogens if combined with outer membrane disruptors [10]. To ascertain the potentiation range of peptides **6**, **10**, **14**, and **19** checkerboard assays with rifampicin, novobiocin, and vancomycin were performed. PMBN was also included to serve as a benchmark and to allow for comparison to other synergists described in literature.

In addition to investigating a broader panel of Gram-positive antibiotics, we were also curious to see how general the synergistic activity of peptides **6**, **10**, **14**, and **19** is against different Gram-negative pathogens. In the initial synergy assays performed the peptides were screened against the indicator strain *E. coli* BW25113. In the next phase of our study we selected a broader panel of Gram-negative bacteria selected from the WHO priority pathogen list. Specifically, we studied the capacity of peptides **6**, **10**, **14**, and **19** to enhance the activity of rifampicin against a range of *E. coli* strains including *mcr*-positive polymyxin-resistant isolates and strains of *A. baumannii*, *P. aeruginosa*, and *Klebsiella pneumoniae*.

#### 2.3.1. Synergy with Rifampicin, Novobiocin, and Vancomycin

**Figure 4:**
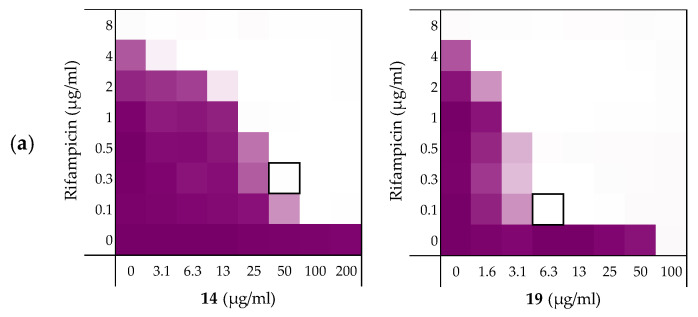

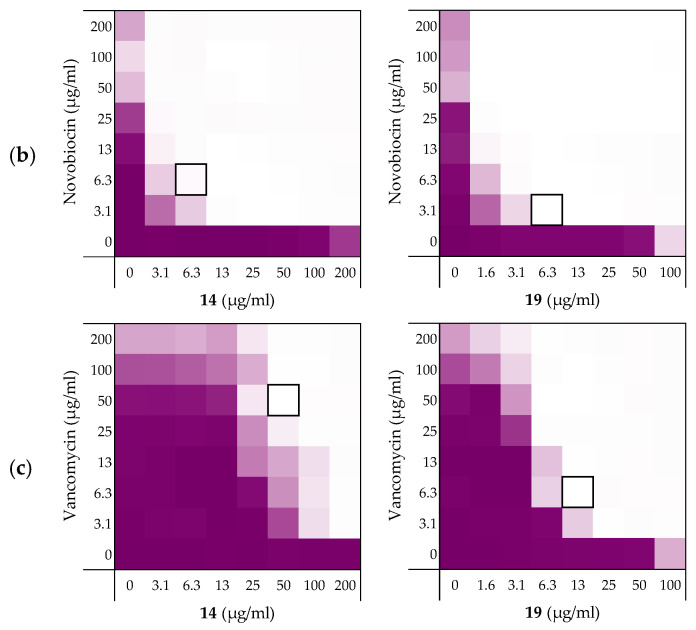
Checkerboard assays of the peptides **14** and **19** in combination with (**a**) rifampicin; (**b**) novobiocin; (**c**) vancomycin versus *E. coli* BW25113. In each case the bounded box in the checkerboard assays indicates the combination of peptide and antibiotic resulting in the lowest FICi (see Table 3). OD_600_ values were measured using a plate reader and transformed to a gradient: purple represents growth, white represents no growth. Checkerboard assays of peptide **6**, **10,** and PMBN in combination with rifampicin, novobiocin and vancomycin are available in the Supplementary data, Figures S2, S5, and S6.

As noted above (Section 2.1), the MIC of rifampicin against *E. coli* BW25113 was established to be 8 µg/mL. By comparison, novobiocin and vancomycin showed no antimicrobial activity against the same strain at concentrations as high as 200 µg/mL. However, when these antibiotics were combined with peptides **6**, **10**, **14**, and **19** a clear synergistic effect was observed in all cases. As noted above, peptides **14** and **19** demonstrated the most potent synergy when combined with erythromycin (Table 2). This effect was largely maintained when **14** and **19** were tested with rifampicin, novobiocin, and vancomycin (Figure 4 and Supplementary data Figures S5 and S6). Table 3 provides an overview of the FICi values obtained for peptides **6**, **10**, **14**, and **19** in combination with these antibiotics. In general, peptide **19** was found to be the most potent synergist and notably was found to be even more effective than PMBN in potentiating the activity of both novobiocin and vancomycin against the indicator strain.

#### 2.3.2. Synergy towards Other *E. coli* Strains Including MCR-Positive Clinical Isolates

**Figure 5:**
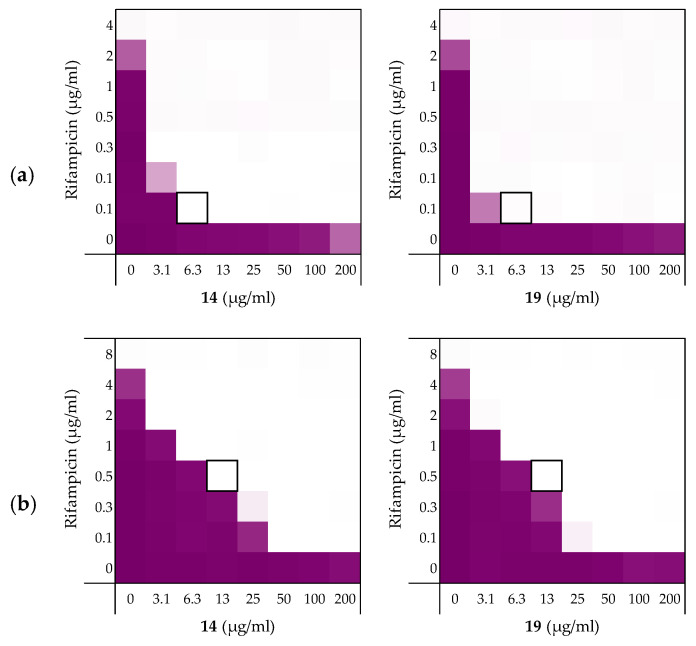

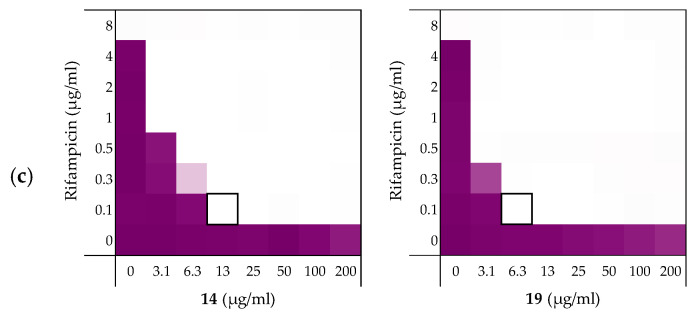
Checkerboard assays of the peptides **14** and **19** in combination with rifampicin versus: (**a**) *E. coli* ATCC25922; and MCR-positive isolates (**b**) *E. coli* EQASmcr-1 and (**c**) *E. coli* EQASmcr-3. In each case the bounded box in the checkerboard assays indicates the combination of peptide and antibiotic resulting in the lowest FICi (see Table 4). OD_600_ values were measured using a plate reader and transformed to a gradient: purple represents growth, white represents no growth. Checkerboard assays of peptide **6** and **10** of the strains shown above and all checkerboard assays of *E. coli* W3110, mcr-1, and EQASmcr-2 are available in the Supplementary data, Figure S7–S12.

Next, the synergistic activity of peptides **6**, **10**, **14**, and **19** in combination with rifampicin was tested against *E. coli* strains ATCC25922 and W3110. These strains were selected given that *E. coli* ATCC25922 has a smooth LPS layer, while *E. coli* W3110 lacks the O-antigen, giving it a rough LPS layer similar to *E. coli* BW25113 [41,42,43]. The susceptibility of Gram-negative bacteria to antibiotics is known to be related to their LPS structure and we therefore set out to assess whether this might affect the efficacy of the synergists as well [44]. While the four peptides exhibited MICs of 200 µg/mL or above against the ATCC25922 strain (see Supplementary data, Table S7), all were found to be potent synergists (Table 4 and Figure 5a). Interestingly, the ATCC25922 strain was found to be more susceptible to these synergistic effects than the BW25113 indicator strain used in our initial screens (Table 4). The results obtained with the W3110 strain provided an interesting contrast: while peptides **6** and **10** exhibited some inherent antimicrobial activity, neither showed any ability to synergize with rifampicin (see Table 4 and Supplementary data, Table S8). Peptides **14** and **19**, however, exhibited potent synergistic activity in combination with rifampicin with peptide **19** resulting in FICi values equal-to or lower than those obtained with the *E. coli* BW25113 indicator strain.

To examine the impact of structurally modified LPS on the synergistic activity of peptides **6**, **10**, **14**, and **19**, the screening was continued with *E. coli* clinical isolates bearing *mcr-1*, *mcr-2* and *mcr-3* genotypes known to confer polymyxin resistance. Specifically, *mcr*-positive bacteria encode for a phosphoethanolamine transferase that modifies the structure of lipid A leading to a loss of activity for polymyxin antibiotics [45,46]. Synergy was confirmed for all *mcr*-positive strains with EQASmcr-1 and EQASmcr-3 shown to be most susceptible to synergy (Figure 5b,c, Table 4 and see Supplementary data, Tables S9–S12). Interestingly, potent synergy was observed for all four peptides with rifampicin indicating that the structurally modified LPS present in these strains does not interfere with the synergistic activity of peptides **6**, **10**, **14**, and **19**.

#### 2.3.3. Synergy towards *A. baumannii*, *K. pneumoniae*, and *P. aeruginosa*

After establishing the synergistic potential of peptides **6**, **10**, **14**, and **19** in combination with rifampicin against several *E. coli* strains, we turned our attention to other Gram-negative pathogens. For this part of the study we elected to use laboratory strains *A. baumannii* ATCC17978, *K. pneumoniae* ATCC13883, and *P. aeruginosa* ATCC27853. Rifampicin was again used as the companion antibiotic and we began by establishing its activity against these strains. While a relatively low MIC of 2 µg/mL was measured for rifampicin against the *A. baumannii* ATCC17978 strain, a much lower activity was found against *K. pneumoniae* ATCC13883 and *P. aeruginosa* ATCC27853 where the MICs measured were 32 µg/mL and 16 µg/mL respectively (see Supplementary data, Tables S13–S15).

**Figure 6:**
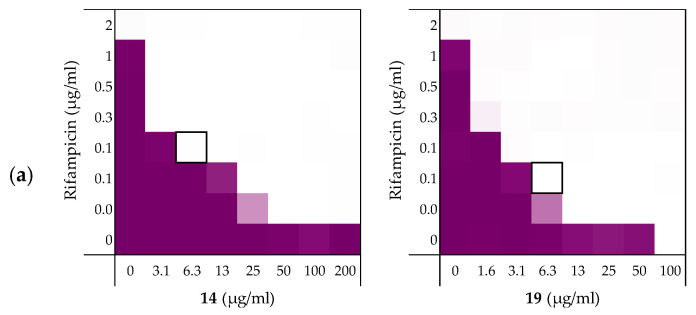

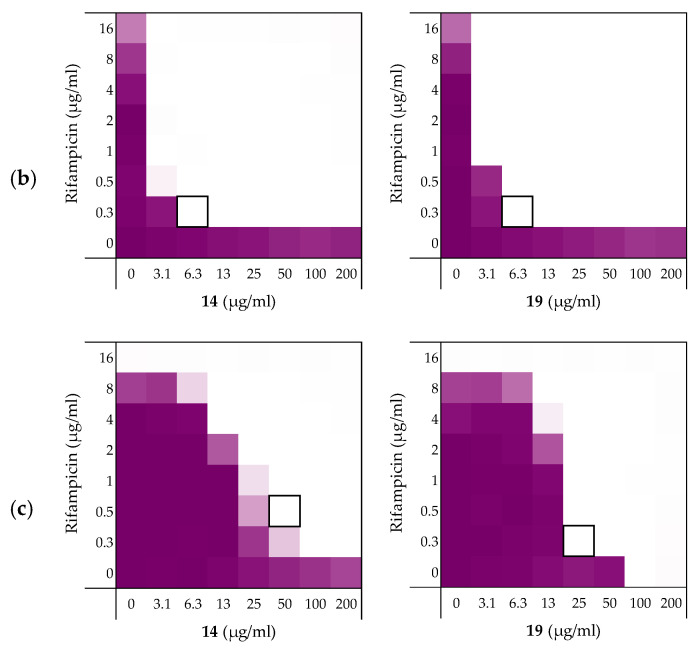
Checkerboard assays for peptides **14** and **19** in combination with rifampicin versus different Gram-negative pathogens: (**a**) *A. baumannii* ATCC17978, (**b**) *K. pneumoniae* ATCC13883, (**c**) *P. aeruginosa* ATCC27853. In each case the bounded box in the checkerboard assays indicates the combination of peptide and antibiotic resulting in the lowest FICi (see Table 5). OD_600_ values were measured using a plate reader and transformed to a gradient: purple represents growth, white represents no growth. Checkerboard assays of peptides **6** and **10** of the strains shown above are available in the Supplementary data, Figures S13–S15.

As illustrated by checkerboard assays of **14** and **19** in Figure 6, all four peptides exhibited potent synergy against the *A. baumannii* and *K. pneumoniae* strains (Table 5). By comparison, significantly less synergy was observed with the *P. aeruginosa* strain with peptide **14** displaying the most potent synergistic activity. The results obtained with *A. baumannii* and *K. pneumoniae* were more in line with our previous findings where again, peptides **14** and **19** resulted in the most potent synergistic combinations with rifampicin. Impressively, a FICi of only 0.023 was detected for both peptides with the *K. pneumoniae* strain tested.

### 2.4. Mechanistic Studies

**Figure 7:**
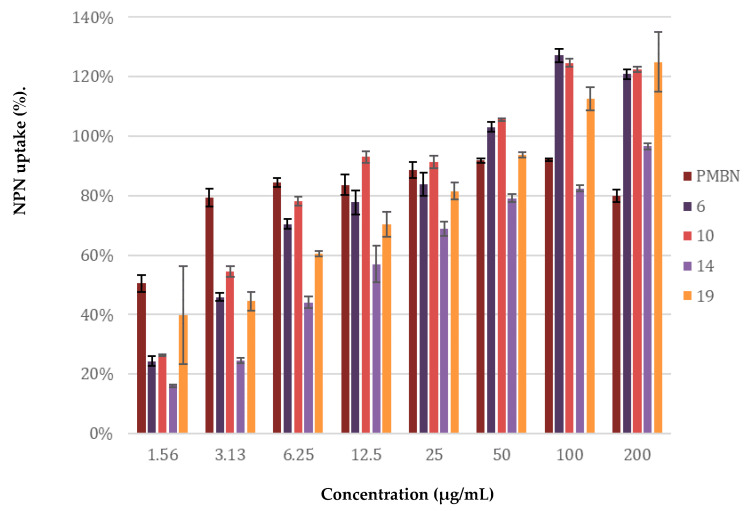
Permeabilization assay of *E. coli* BW25113 using *N*-Phenyl-1-naphthylamine as fluorescent probe. The read-out was performed after 60 min of incubation using a plate reader with λ_ex_ 355 nm and λ_em_ 420 nm. The *N*-phenyl-napthalen-1-amine (NPN) uptake values shown are relative to the uptake signal obtained upon treating the cells with 100 µg/mL colistin as previously reported [48]. Error bars represent the standard deviation based on n = 3 technical replicates. A read-out was also performed after 10 min of incubation (see Supplementary data, Figure S16).

The potentiation of antibiotics like erythromycin, rifampicin, novobiocin and vancomycin against Gram-negative bacteria is generally attained by disruption of the OM as previously described for PMBN [10]. The potent synergy observed for peptides **6**, **10**, **14**, and **19** with these antibiotics, coupled with the finding that the peptides are largely non-hemolytic, points to a mode of action involving selective permeabilization of the Gram-negative OM. To further investigate this hypothesis, a permeabilization assay using *N*-phenyl-napthalen-1-amine (NPN) on *E. coli* BW25113 was performed. This assay enables monitoring of OM disruption based upon the ability of NPN to enter the phospholipid layer which in turn results in a detectable increase in fluorescence [47]. As illustrated in Figure 7, a clear dose-dependent effect was observed for peptides **6**, **10**, **14**, **19**. Taken along as a benchmark, PMBN was found to induce ca. 80% OM permeabilization at a concentration of 3.13 µg/mL. By comparison, the peptides matched or surpassed this effect at the higher concentrations tested.

### 2.5. Stereochemical Studies 

We next set out to probe the stereochemical parameters governing the OM disrupting activity of peptides **6**, **10**, **14**, and **19**. The result of the LPS competition assay with peptide **6** (described above in Section 2.1) as well as the published NMR studies on related thrombin-derived C-terminal peptides [26], suggest that these peptides interact with LPS. At the core of the LPS structure is the bacterial phospholipid lipid A. Given that lipid A is a chiral biomolecule, we next prepared a series of stereochemical analogues of peptide **6** and characterized the impact on synergistic activity. These analogues included the all d-amino acid enantiomeric species **20**, the l-amino acid inverso peptide **21** and the all d-amino acid retro-inverso variant **22**. 

**Figure 8:**
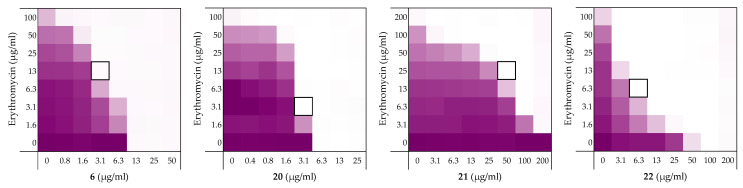
Checkerboard assays of the peptides **6**, and stereochemical analogues **20**–**22** in combination with erythromycin versus *E. coli* BW25113. In each case the bounded box in the checkerboard assays indicates the combination of peptide and antibiotic resulting in the lowest FICi (see Table 1). OD_600_ values were measured using a plate reader and transformed to a gradient: purple represents growth, white represents no growth. An overview of all checkerboard assays with erythromycin can be found in the Supplementary data, Figure S1.

Peptide **6** and enantiomer **20** were both found to exhibit appreciable inherent antimicrobial activity against *E. coli* BW25113, with MICs of 12.5 µg/mL and 6.25 µg/mL respectively (Table 6). Inversion of these peptides to give **21** and **22** led to a significant loss of antibacterial activity in both cases with MICs of >200 µg/mL and 100 µg/mL respectively. Checkerboard assays were next performed with erythromycin as the companion antibiotic using *E. coli* BW25113 as the indicator strain (Figure 8). Interestingly, the enantiomeric peptide **20** was found to exhibit no synergetic activity suggesting that the synergy observed for **6** is indeed stereospecific. Interestingly, the l-inverso peptide **21** did exhibit moderate synergistic activity, however, the d-retro-inverso peptide **22** was a much more potent synergist. Give that retro-inverso peptides can assume a side chain topology similar to that of the parent l-peptide [49], these finding further support the stereospecific mechanism of peptide **6**. Similar results were obtained upon repeating the synergy assays for peptides **6**, **20**–**22** with rifampicin as the companion antibiotic (see Supplementary data, Figure S2 and Table S2).

## 3. Discussion

The LPS-binding potential of the TCPs sparked our interest in these peptides as potential synergists. Indeed, the synergistic activity of peptides **1**, **2**, and **3** validated this hypothesis (See Table 2). Notably, this represents the first demonstration of the synergistic activity for these peptides even though their antimicrobial activity has been well-studied [26,35,36,50,51,52]. Peptide **1** exhibits synergy comparable to PMBN (Table 1). Amidation of the C-terminus of peptide **1** gave peptide **6** and led to a significant enhancement of inherent antibiotic activity, an effect also known for other antimicrobial peptides [53]. Most importantly, peptide **6** maintained synergistic activity leading to the lowest MSC and was therefore selected as a lead for further investigation. 

While C-terminal amidation impacts the overall charge in peptide **6**, LPS-binding is still maintained as evidenced by the results of an LPS competition assay (Supplementary data, Figure S4 and Table S4). Moreover, stereochemical studies with peptide **6** suggests that synergistic activity is indeed stereospecific: loss of synergistic activity was observed for the mirror image of peptide **6**, d-peptide **20** (see Table 6). Similarly, in literature the mirror image of PMBN was described to have no synergistic activity [54]. Notable, however, is the finding that retro-inverso peptide **22** displays potent synergistic activity, in line with expectations given that the retro-inverso analogue features a topology similar to parent peptide **6 [49]**. 

Another indication of a mechanism involving LPS-binding comes from the antimicrobial activity observed for peptide **6** against wild-type and *mcr-1* strains of *E. coli*: while MIC values of 12.5 µg/mL and 6.25 µg/mL where measured against the reference BW25113 and W3110 strains, respectively, in the case of the *mcr-1,2,3* clinical isolates tested the MICs were much higher (50–100 µg/mL, see Supplementary data, Tables S2, S8–S12). A similar trend is also observed for the established LPS-binding polymyxin class of antibiotics [45,46]. Interestingly, the synergistic activity of peptide **6**, and the alanine-scan derived analogues **10**, **14**, and **19** is well retained against *mcr*-type stains which is not the case for PMBN (Table 4) [31]. 

The alanine scan provided insight into the roles of each amino acid in peptide **6** and resulted in the identification of three potent synergists: peptide **10**, **14**, and **19** (Table 2). All three peptides potentiated erythromycin, rifampicin, novobiocin, and vancomycin (Table 2 and Table 3). Peptide **19** was on par with PMBN and results in lower or equal FICi’s of synergists recently described in literature [31,33]. Impressively, the potentiation of rifampicin by peptide **14** and **19** was also seen against multiple *E. coli* strains including the *mcr*-clinical isolates, *K. pneumoniae*, *P. aeruginosa*, and *A. baumannii* also with very low FICi values (Table 4 and Table 5). By comparison, peptide **10** displayed a slightly lower synergistic activity than peptide **14** and **19**, but was still equal to or better than peptide **6**. Interestingly, peptide **14** features the substitution of Ala for Trp, a residue often associated with membrane binding and antimicrobial activity [55,56,57,58]. Indeed, a significant loss of inherent antimicrobial activity is observed for peptide **14** relative to **6**. However, the finding that peptide **14** retains potent synergy suggests the Trp is not key for synergistic activity or OM permeabilization (Figure 7). 

What is also clear from the alanine scan is the essentiality of the lysine side-chains, not only for maintaining synergy, but also for limiting hemolytic activity (Table 2). Comparable findings have been reported for PMB and PMBN which contain several positively charged Dab resides and replacing them with uncharged amino acids leads to a loss of antimicrobial activity for PMB and synergistic activity for PMBN [59]. 

In summary, the peptides investigated in this study were found to exhibit potent and targeted synergy with multiple Food and Drug Administration (FDA)-approved Gram-positive-specific antibiotics. Importantly, this synergy was demonstrated against a range of Gram-negative species including *mcr*-resistant strains. The selective OM disrupting properties of these peptides and their potent synergy highlights the potential for such compounds to expand the number of antibiotic classes that can be effectively employed to kill Gram-negative bacteria. 

## 4. Materials and Methods

### 4.1. Manual Peptide Synthesis for Carboxylic Acid C-terminus 

Chlorotrityl resin (5.0 g, 1.60 mmol·g^−1^) was loaded with Fmoc-Ile-OH (1, 5) or Fmoc-Glu(OtBu)-OH (2-4). Resin loading was determined to be 0.44–0.57 mmol·g^−1^. Linear peptide encompassing the first amino acid until the last amino acid were assembled manually via standard Fmoc solid-phase peptide synthesis (SPPS) (resin bound AA:Fmoc-AA:BOP:DiPEA, 1:4:4:8 molar eq.) on a 0.1 mmol scale. DMF was used as solvent and Fmoc deprotections were carried out with piperidine:DMF (1:4 v:v). Amino acid side chains were protected as follows: tBu for Asp/Glu, Trt for Asn/Gln, Boc for Lys/Trp, Pbf for Arg. Following coupling and Fmoc deprotection of the final amino acid, N-terminal acylation was achieved for peptide 5 by coupling Ac_2_O using the same coupling conditions used for SPPS. The resin-bound peptides were next washed with CH_2_Cl_2_ and subsequently treated with TFA:TIS:H_2_O (95:2.5:2.5, 10 mL) for 90 min. Resin beads were filtered off and the reaction mixture was added to cold MTBE:hexanes (1:1) and the resulting precipitate washed once more with MTBE:hexanes (1:1). The crude peptide was lyophilized from tBuOH:H_2_O (1:1) and purified with reverse phase HPLC. Pure fractions were pooled and lyophilized to yield the desired linear peptides as white powders, typically in 10–20 mg quantities. For peptide characterization and analysis see Supplementary data, Table S16 and pages S26–S28.

### 4.2. Automated Peptide Synthesis for C-terminal Amides

Rink Amide resin (150 mg, 0.684 mmol·g^−1^) was loaded into the reaction vessel of the CEM liberty blue peptide synthesizer for a 0.1 mmol scale. Linear peptides 6–22 were assembled using microwave irradiation at 90 C (resin bound AA:Fmoc-AA:DIC:Oxyma, 1:5:5:5 molar eq.). DMF was used as solvent and Fmoc deprotections were carried out with piperidine:DMF (1:4, v:v). Amino acid side chains were protected as follows: tBu for Asp/Glu, Trt for Asn/Gln, Boc for Lys/Trp, Pbf, for Arg. Following coupling and Fmoc deprotection of the final amino acid, N-terminal acylation was achieved for peptide 7 by coupling Ac_2_O using microwave irradiation at 90 C. The linear peptides were removed from the reaction vessel, washed with DCM and directly treated with TFA:TIS:H2O (95:2.5:2.5, 10 mL) for 90 min. Resin beads were filtered off and the reaction mixture was added to cold MTBE:hexanes (1:1) and the resulting precipitate washed once more with MTBE:hexanes (1:1). The crude peptides was lyophilized from tBuOH:H2O (1:1) and purified with reverse phase HPLC. Pure fractions were pooled and lyophilized to yield the desired linear peptides as white powders, typically in 20–60 mg quantities. For peptide characterization and analysis see Supplementary data, Table S16 and pages S28–S36.

### 4.3. Antimicrobial Assays 

All peptides were screened for antimicrobial activity against *E. coli* BW25113, *E. coli* ATCC25922, and *E. coli* W3110. A select group of the peptides was further tested against *E. coli* mcr-1, *E. coli* EQASmcr-1 (EQAS 2016 412016126), *E. coli* EQASmcr-2 (EQAS 2016 KP37), *E. coli* EQASmcr-3 (EQAS 2017 2013-SQ352), *K. pneumoniae* ATCC13883, *P. aeruginosa* ATCC27853, and *A. baumannii* ATCC17978. The antimicrobial assay was performed according to CLSI guidelines. Bacteria were plated out directly from their glycerol stocks on blood agar plates, incubated overnight at 37 °C, and then kept in the fridge. The blood agar plates were only used for 2 weeks and then replaced.

### 4.4. Minimal Inhibitory Concentration (MIC) Assay 

One colony from a blood agar plates was inoculated in Lysogeny Broth (LB) at 37 °C until a 0.5 optical density at 600 nm (OD_600_) was reached (compared to the sterility control of LB). The bacterial suspension was diluted in fresh LB to 2.0 × 10^6^ CFU/mL. The serial dilutions were prepared in polypropylene microtiter plates: a stock of the test compounds was prepared with a 2x final concentration in LB. Moreover, 100 µL of the stock was added to the wells of the top row of which 50 µL was used for the serial dilution. The bottom row of each plate was used as the positive (50 µL of LB) and negative controls (100 µL of LB) (6 wells each). Furthermore, 50 µL of the 2.0 × 10^6^ CFU/mL bacterial stock was added to each well except for the negative controls, adding up to a total volume of 100 µL per well. The plates were sealed with a breathable seal and incubated for 20 h at 37 °C and 600 rpm. The MIC was visually determined after centrifuging the plates for 2 min at 3000 rpm. 

### 4.5. Checkerboard Assays 

Two 96-well plates were required per compound. A serial dilution of the compound was performed on two plates from row A–G using with LB as medium. Row H was filled with 25 µL medium. On a separate plate a negative control (8 wells, 100 μL medium) was prepared. For all concentrations of antibiotic a specific stock was prepared. 25 µL medium was added to the 0 µg/mL antibiotic columns (plate I, column 1–3). Of each antibiotic stock 25 µL was added to three columns to obtain triplicates for every condition. Both plates contained 50 µL in each well to which 50 µL bacterial stock was added (see the protocol of the MIC assays) and the plates were sealed. After incubation for 20 h at 37 °C while shaking, the breathable seals were removed and the density of the bacterial suspensions measured at 600 nm (OD_600_) using a Tecan Spark plate reader. The OD_600_ values were transformed to a 2D gradient with the negative control as minimum and the positive control (wells G1, G2, and G3) as maximum in excel. The FICi was calculated using Equation (1) with a FICi ≤ 0.5 indicating synergy [37].

FICi = (MSC_a_/MIC_a_) + (MSC_b_/MIC_b_), (1)

Calculation of the FICi: MSC_a_ = concentration of the synergist in combination with the antibiotic that results in the lowest FICi. MIC_a_ = MIC of the antibiotic without synergist (row G). MSC_b_ = concentration of the synergist in combination with the antibiotic that results in the lowest FICi. MIC_b_ = MIC of the synergist itself (Column 1–3, plate I).

### 4.6. Hemolysis Assay 

Defibrinated sheep blood (Thermo Fisher Scientific) and a phosphate-buffered saline (PBS) buffer containing 0.002% Tween20 were used in this assay. The red blood cells were washed with buffer followed by centrifugation (400 g for 15 min at 4 °C). The red blood cells were then normalized to obtain a positive control read-out around 2.5 at 415 nm to stay in the linear range with the maximum sensitivity. A serial dilution of the compounds (200–1.56 µg/mL, 75 µL) was prepared in a 96-well plate. Each plate contained a positive control (0.1% Triton X-100 final concentration, 75 µL) and the negative control (buffer, 75 µL). After addition of normalized blood cells (75 µL), the plates were incubated for 20 h at 37 °C at 500 rpm. The plates were centrifuged (800× *g* for 5 min at room temperature). For the next step flat-bottom polystyrene plates were prepared with 100 µL buffer in each well. Aliquots of 25 µL supernatant from the centrifuged plates were then transferred to their respective wells in the flat-bottom plate. Absorbance values where measured at 415 nm and corrected for background (negative control) and transformed to a percentage hemolysis relative to the positive control. 

### 4.7. LPS Competition Assay 

The same protocol as the MIC assay was used to prepare the serial dilution of the compounds in 96-wells plates in duplicate resulting in two identical plates. A serial dilution of colistin was taken along as a control. The inoculation and preparation of the bacteria stock was performed as described for the MIC assay. The stock of bacteria was then split into two stocks. LPS (1 mg/mL final concentration) was added to one of the stocks and added to one of the duplicate plates as described in the MIC assay. The normal bacteria stock was added to the remaining plate as described in the MIC assay. The plates were sealed with a breathable seal and incubated for 20 h at 37 °C and 600 rpm after which the MIC was visually determined.

### 4.8. Membrane Permeability Assay Using N-phenylnaphthalen-1-amine (NPN)

This assay was adapted from a protocol described in literature [47,48]. Bacteria were inoculated overnight at 37 °C in LB, diluted the next day 50× in LB and grown to OD_600_ of 0.5. The bacterial suspension was then centrifuged for 10 min at 1000× *g* at 25 °C. The pellet of bacteria was resuspended in 5 mM HEPES buffer containing 20mM glucose to a final concentration of OD_600_ of 1.0. The compounds were serial diluted (25 µL) in triplicate in a black ½ area clear-bottom 96-well plate. Moreover, 100 µg/mL final concentration of colistin in triplicate served as the positive control. Three wells were filled with 25 µL buffer to serve as the negative control. Additional controls of the compounds were made in triplicate using 25 µL of the highest concentration to detect interactions of the compounds with NPN in the absence of bacteria. A stock of 0.5 mM of NPN in acetone was prepared and diluted 12.5× in the buffer; 25 µL of the NPN solution was added to each well; 50 μL of the 1.0 OD_600_ bacterial stock was then added to each well except for the controls of the compounds with NPN. To these wells, 50 µL of buffer was added. After 10 and 60 min, the plate was measured using Tecan plate reader with λex 355 nm ±20 nm and λem 420 nm ± 20 nm. The fluorescence values obtained were then transformed into a NPN uptake percentage using the following Equation (2):

NPN uptake (%) = (F_obs_ − F_0_)/(F_100_ − F_0_) × 100%, (2)

Equation (2) consists of an observed value of fluorescence (F_obs_), which is corrected for background using the negative control (F_0_). This value is divided by the positive control corrected for background (F_100_–F_0_) and multiplied by 100% to obtain the percentage [60].

## Figures and Tables

**Table 1:** Overview of peptide sequences, synergistic activity, and hemolytic activity.

	Peptide Sequence	MIC ^a^	MSC_peptide_^b^	FICi ^c^	Hemolysis ^d^
**1**	H_2_N-VFRLKKWIQKVI-OH	200	25	0.188	0%
**2**	H_2_N-HVFRLKKWIQKVIDQFGE-OH	200	50	0.375	9%
**3**	H_2_N-FYTHVFRLKKWIQKVIDQFGE-OH	200	50	0.500	43%
**4**	H_2_N-GKYGFYTHVFRLKKWIQKVIDQFGE-OH	>200	12.5	>0.5	70%
**5**	Ac-VFRLKKWIQKVI-OH	>200	50	0.156	0%
**6**	H_2_N-VFRLKKWIQKVI-CONH_2_	12.5	3.13	0.313	4%
**7**	Ac-VFRLKKWIQKVI-CONH_2_	50	12.5	0.266	5%
**PMBN**		>200	25	0.125	-

^a^ Minimum inhibitory concentration (µg/mL). ^b^ Minimum synergistic concentration (µg/mL). ^c^ Synergy defined as an FICi ≤ 0.5 [37]. ^d^ Hemolysis was determined after a 20 h incubation of the compounds (200 µg/mL) with defibrinated sheep blood (see Supplementary data, Figure S3 and Table S3).

**Table 2:** Overview of the Ala-scan peptide sequences, antimicrobial, synergistic, and hemolytic activity. Minimal Inhibitory Concentration (MIC) and FICi values were obtained from the checkerboard assay shown in Figure 3.

	Peptide Sequence	MIC ^a^	FICi ^b^	Hemolysis ^c^
**6**	H_2_N-VFRLKKWIQKVI-NH_2_	12.5	0.313	4%
**8**	H_2_N-**A**FRLKKWIQKVI-NH_2_	12.5	0.188	2%
**9**	H_2_N-V**A**RLKKWIQKVI-NH_2_	50	0.125	0%
**10**	H_2_N-VF**A**LKKWIQKVI-NH_2_	100	0.141	4%
**11**	H_2_N-VFR**A**KKWIQKVI-NH_2_	200	0.188	1%
**12**	H_2_N-VFRL**A**KWIQKVI-NH_2_	25	>0.5	30%
**13**	H_2_N-VFRLK**A**WIQKVI-NH_2_	25	>0.5	19%
**14**	H_2_N-VFRLKK**A**IQKVI-NH_2_	>200	0.078	1%
**15**	H_2_N-VFRLKKW**A**QKVI-NH_2_	100	0.188	1%
**16**	H_2_N-VFRLKKWI**A**KVI-NH_2_	25	0.188	4%
**17**	H_2_N-VFRLKKWIQ**A**VI-NH_2_	12.5	>0.5	21%
**18**	H_2_N-VFRLKKWIQK**A**I-NH_2_	50	0.125	2%
**19**	H_2_N-VFRLKKWIQKV**A**-NH_2_	100	0.094	1%

^a^ MSC data can be found in Supplementary data, Table S1. ^b^ Synergy defined as an FICi ≤ 0.5 [37]. ^c^ Hemolysis determined after a 20 h incubation of the compounds (200 µg/mL) with defibrinated sheep blood. (See Supplementary data, Figure S3 and Table S3).

**Table 3:** FICi values of peptides **6**, **10**, **14**, and **19** against *E. coli* BW25113 in combination with “Gram-positive-specific” antibiotics rifampicin, novobiocin, and vancomycin ^a^.

Peptides	Rifampicin	Novobiocin	Vancomycin
6	0.156	0.188	0.188
**10**	0.141	0.078	0.156
**14**	0.141	0.031	0.250
**19**	0.078	0.039	0.078
**PMBN**	0.063	0.047	0.156

^a^ MIC and MSC data can be found in the Supplementary data, Tables S2, S5, and S6.

**Table 4:** FICi values of peptides 6, 10, 14, and 19 in combination with rifampicin against different *E. coli* strains including mcr-resistant strains ^a^.

Pathogen	6	10	14	19
*E. coli* BW25113	0.156	0.141	0.141	0.078
*E. coli* ATCC25922^TM^	0.047	0.031	0.031	0.031
*E. coli* W3110	>0.5 ^b^	>0.5 ^a^	0.188	0.078
*E. coli* mcr-1	0.141	0.078	0.125	0.125
*E. coli* EQASmcr-1	0.078	0.078	0.094	0.094
*E. coli* EQASmcr-2	0.094	0.141	0.094	0.125
*E. coli* EQASmcr-3	0.078	0.078	0.047	0.031

^a^ MIC and MSC data can be found in the Supplementary data, Tables S7–S12. ^b^ Synergy is defined in literature as a FICi ≤ 0.5 [37].

**Table 5:** FICi values of peptides **6**, **10**, **14**, and **19** in combination with rifampicin against different Gram-negative pathogens ^a^.

Pathogen	6	10	14	19
*A. baumannii* ATCC17978	0.125	0.125	0.078	0.094
*K. pneumoniae* ATCC13883	0.063	0.063	0.023	0.023
*P. aeruginosa* ATCC27853	>0.5 ^b^	0.250	0.156	0.266

^a^ MIC and MSC data can be found in the Supplementary data, Tables S13–S15. ^b^ Synergy is defined in literature as a FICi ≤ 0.5 [37].

**Table 6:** Overview of the TCPs peptide sequence, synergistic and hemolytic activity.

	Peptide Sequence	MIC	MSC_peptide_	FICi ^a^	Hemolysis ^b^
**6**	H_2_-VFRLKKWIQKVI-NH_2_	12.5	3.13	0.313	4%
**20**	H_2_N-vfrlkkwiqkvi-NH_2_	6.25	3.13	>0.5 ^a^	3%
**21**	H_2_N-IVKQIWKKLRFV-NH_2_	>200	50	0.250	2%
**22**	H_2_-ivkqiwkklrfv-NH_2_	100	6.25	0.094	3%

^a^ Synergy defined as an FICi ≤ 0.5 [37].^b^ Hemolysis determined after a 20 h incubation of the compounds (200 µg/mL) with defibrinated sheep blood (see Supplementary data, Figure S3 and Table S3).

## Data Availability

Full materials, synthesis, and purification methods and characterization data for all compounds synthesized are available online. All supplementary biological data are also included and mentioned under Supplementary Materials (above).

## Supplementary Materials

The following are available online, Figure S1: Checkerboard assays of the peptides **1**–**22** and PMBN in combination with erythromycin versus *E. coli* BW25113. Figure S2: Checkerboard assays of the peptides **1**–**22** and PMBN in combination with rifampicin versus *E. coli* BW25113. Figure S3: Hemolytic activity of peptides **1**–**22** (200 µg/mL). Figure S4: LPS competition assay of **6** with *E. coli* BW25113. Figure S5: Checkerboard assays of the peptides **6**, **10**, **14**, **19,** and PMBN in combination with novobiocin versus *E. coli* BW25113. Figure S6: Checkerboard assays of the peptides **6**, **10**, **14**, **19,** and PMBN in combination with vancomycin versus *E. coli* BW25113. Figure S7: Checkerboard assays of the peptides **6**, **10**, **14**, and **19** in combination with rifampicin versus *E. coli* ATCC25922. Figure S8: Checkerboard assays of the peptides **6**, **10**, **14**, and **19** in combination with rifampicin versus *E. coli* W3110. Figure S9: Checkerboard assays of the peptides **6**, **10**, **14**, and **19** in combination with rifampicin versus *E. coli* mcr-1. Figure S10: Checkerboard assays of the peptides **6**, **10**, **14**, and **19** in combination with rifampicin versus *E. coli* EQASmcr-1. Figure S11: Checkerboard assays of the peptides **6**, **10**, **14**, and **19** in combination with rifampicin versus *E. coli* EQASmcr-2. Figure S12: Checkerboard assays of the peptides **6**, **10**, **14**, and **19** in combination with rifampicin versus *E. coli* EQASmcr-3. Figure S13: Checkerboard assays of the peptides **6**, **10**, **14**, and **19** in combination with rifampicin versus *A. baumannii* ATCC17978. Figure S14: Checkerboard assays of the peptides **6**, **10**, **14**, and **19** in combination with rifampicin versus *K. pneumoniae* ATCC13883. Figure S15: Checkerboard assays of the peptides **6**, **10**, **14**, and **19** in combination with rifampicin versus *P. aeruginosa* ATCC27853. Figure S16: Permeabilization assay of *E. coli* BW25113 with peptides **6**, **10**, **14**, and **19,** and PMBN using *N*-Phenyl-1-naphthylamine as fluorescent probe. Table S1: Synergetic data of peptides **1**–**22** and PMBN of the checkerboard assays with rifampicin in Figure S1. Table S2: Synergetic data of peptides **1-22** and PMBN of the checkerboard assays with rifampicin in Figure S2. Table S3: Hemolytic activity of peptides **1-22** (200 µg/mL). Table S4: Overview of LPS competition results of peptide **6**. Table S5: Synergetic data of peptides **6**, **10**, **14**, **19,** and PMBN with novobiocin of the checkerboard results displayed in Figure S5. Table S6: Synergetic data of peptides **6**, **10**, **14**, and **19** and PMBN with vancomycin of the checkerboard results displayed in Figure S6. Table S7: Synergistic data of peptides **6**, **10**, **14**, and **19** of the checkerboard results for *E. coli* ATCC25922 with rifampicin displayed in Figure S7. Table S8: Synergistic data of peptides **6**, **10**, **14**, and **19** of the checkerboard results for *E. coli* W3110 with rifampicin displayed in Figure S8. Table S9: Synergistic data of peptides **6**, **10**, **14**, and **19** of the checkerboard results for *E. coli* mcr-1 with rifampicin displayed in Figure S9. Table S10: Synergistic data of peptides **6**, **10**, **14**, and **19** of the checkerboard results for *E. coli* EQASmcr-1 with rifampicin displayed in Figure S10. Table S11: Synergistic data of peptides **6**, **10**, **14**, and **19** of the checkerboard results for *E. coli* EQASmcr-2 with rifampicin displayed in Figure S11. Table S12: Synergistic data of peptides **6**, **10**, **14**, and **19** of the checkerboard results for *E. coli* EQASmcr-3 with rifampicin displayed in Figure S12. Table S13: Synergistic data of peptides **6**, **10**, **14**, and **19** of the checkerboard results for *A. baumannii* ATCC17978 with rifampicin displayed in Figure S13. Table S14: Synergistic data of peptides **6**, **10**, **14**, and **19** of the checkerboard results for *K. pneumoniae* ATCC13883 with rifampicin displayed in Figure S14. Table S15: Synergistic data of peptides **6**, **10**, **14**, and **19** of the checkerboard results for *P. aeruginosa* ATCC27853 with rifampicin displayed in Figure S15. Table S16: Overview of the HRMS results.

## Sample Availability

Samples of the compounds 1–22 are available from the authors.
