# Supplementary material for: Thrombin-Derived Peptides Potentiate the Activity of Gram-Positive-Specific Antibiotics against Gram-Negative Bacteria
Source: Molecules. 2021 Mar 30;26(7):1954. doi: 10.3390/molecules26071954 (PMC8037310; doi:10.3390/molecules26071954)
Supplement: Supplementary file 1 [file molecules-26-01954-s001.pdf]

Supplementary data

Thrombin-derived peptides potentiate the activity of Gram-positive-specific antibiotics against Gram-negative bacteria

Charlotte M.J. Wesseling [1,†], Thomas M. Wood [1,2,†], Kristine Bertheussen [1,3], Samantha Lok [1] and Nathaniel I. Martin [1,*]

1 Biological Chemistry Group, Institute of Biology Leiden, Leiden University, Leiden, The Netherlands; c.m.j.wesseling@biology.leidenuniv.nl, t.m.wood@biology.leidenuniv.nl, n.i.martin@biology.leidenuniv.nl
2 Department of Chemical Biology & Drug Discovery, Utrecht Institute for Pharmaceutical Sciences, Utrecht University, Utrecht, The Netherlands
3 Bio-organic Synthesis, Leiden Institute of Chemistry, Leiden University, Leiden, The Netherlands
* Correspondence: n.i.martin@biology.leidenuniv.nl
† These authors contributed equally to this work.

Table of content

General notes S3

Synthesis S4

Biological data of peptides **1-22** and PMBN against *E.coli* BW25113
- with erythromycin S6
- with rifampicin S8

Hemolysis assay of peptides **1-22** S10

LPS competition assay of peptide **6** S12

Synergy data of peptides **6**, **10**, **14**, **19**, and PMBN against BW25113
- with novobiocin S13
- with vancomycin S14

Synergy data of peptides **6**, **10**, **14**, and **19** with rifampicin
- *E.coli* ATCC25922 S15
- *E.coli* W3110 S16
- *E.coli* mcr-1 S17
- *E.coli* EQASmcr-1 (=EQAS 2016 412016126) S18
- *E.coli* EQASmcr-2 (=EQAS 2016 KP37) S19
- *E.coli* EQASmcr-3 (=EQAS 2017 2013-SQ352) S20
- *A. baumannii* ATCC17978 S21
- *K. pneumoniae* ATCC13883 S22
- *P. aeruginosa* ATCC27853 S23

Membrane permeability assay using NPN S24

Peptide characterization and analysis S25
- HRMS data S25
- HPLC traces S26

Sources of bacterial strains S37

References S38

General notes

All reagents employed were of American Chemical Society (ACS) grade or finer and were used without further purification unless otherwise stated. For compound characterization HRMS analysis was performed on a Shimadzu Nexera X2 UHPLC system with a Waters Acquity HSS C18 column (2.1 × 100 mm, 1.8 μm) at 30 °C and equipped with a diode array detector. The following solvent system, at a flow rate of 0.5 mL/min, was used: solvent A, 0.1 % formic acid in water; solvent B, 0.1 % formic acid in acetonitrile. Gradient elution was as follows: 95:5 (A/B) for 1 min, 95:5 to 15:85 (A/B) over 6 min, 15:85 to 0:100 (A/B) over 1 min, 0:100 (A/B) for 3 min, then reversion back to 95:5 (A/B) for 3 min. This system was connected to a Shimadzu 9030 QTOF mass spectrometer (ESI ionization) calibrated internally with Agilent's API-TOF reference mass solution kit (5.0 mM purine, 100.0 mM ammonium trifluoroacetate and 2.5 mM hexakis(1H,1H,3H-tetrafluoropropoxy)phosphazine) diluted to achieve a mass count of 10000. Purity of the peptides was confirmed to be ≥ 95% by analytical RP-HPLC using a Shimadzu Prominence-i LC-2030 system with a Dr. Maisch ReproSil Gold 120 C18 column (4.6 × 250 mm, 5 μm) at 30 °C and equipped with a UV detector monitoring at 214 nm. The following solvent system, at a flow rate of 1 mL/min, was used: solvent A, 0.1 % TFA in water/acetonitrile, 95/5; solvent B, 0.1 % TFA in water/acetonitrile, 5/95. Gradient elution was as follows: 95:5 (A/B) for 2 min, 95:5 to 0:100 (A/B) over 13 min, 0:100 (A/B) for 2 min, then reversion back to 95:5 (A/B) over 1 min, 95:5 (A/B) for 2 min. The compounds were purified via preparative HPLC using a BESTA-Technik system with a Dr. Maisch Reprosil Gold 120 C18 column (25 × 250 mm, 10 μm) and equipped with a ECOM Flash UV detector monitoring at 214 nm. The following solvent system, at a flow rate of 12 mL/min, was used: solvent A, 0.1 % TFA in water/acetonitrile 95/5; solvent B, 0.1 % TFA in water/acetonitrile 5/95. Gradient elution was as follows: 95:5 (A/B) for 2 min, 95:5 to 0:100 (A/B) over 30 min, 0:100 (A/B) for 2 min, then reversion back to 95:5 (A/B) over 1 min, 95:5 (A/B) for 2 min.

Peptide synthesis

Automated peptide synthesis. Peptides were synthesized by a microwave-assisted peptide synthesizer (Liberty Blue HT-12, CEM) using the following cycles of deprotection and coupling.

1) Fmoc deprotection: 90 ºC, 80 W, 65 s with 20% piperidine in DMF, 3 mL/deprotection

2) AA coupling: Fmoc-AA-OH (0.2M in 2.5 mL DMF, 5 eq), DIC (1M in 1 mL DM, 10 eq) and Oxyma (1M in 0.5 mL DMF, 5 eq) at 76 ºC, 80 W, 15 s before the temperature was increased to 90 ºC, 80 W for 110s.

Peptide abbreviations

AA	amino acid
Boc	*tert*-butyloxycarbonyl
^{t}Bu	*tert*-butyl
^{t}BuOH	*tert*-butanol
Boc	*tert*-butyloxycarbonyl
BOP	(benzotriazol-1-yloxy)tris(dimethylamino)phosphonium hexafluorophosphate
DIC	*N,N*-Diisopropylcarbodiimide
DiPEA	*N,N*-diisopropylethylamine
DMF	*N,N*-dimethylformamide
Fmoc	Fluorenylmethyloxycarbonyl
HFIP	1,1,1,3,3,3-hexafluoro-2-propanol
MTBE	Methyl *tert*-butyl ether
Oxyma	Ethyl cyanohydroxyiminoacetate
Pbf	2,2,4,6,7-pentamethyldihydrobenzofuran-5-sulfonyl
TIS	triisopropylsilane
Trt	trityl

Synthesis of C-terminal acid peptides.

Chlorotrityl resin was loaded with the first Fmoc-AA-OH (depending on the sequence). Linear peptide encompassing the first AA to the last AA was assembled manually via standard Fmoc solid-phase peptide synthesis (SPPS) (resin bound AA:Fmoc-AA:BOP:DiPEA, 1:4:4:8 molar eq.) on a 0.25 mmol scale. DMF was used as solvent and Fmoc deprotections were carried out with piperidine:DMF (1:4 v:v). Amino acid side chains were protected as follows: ^{t}Bu for Ser/Asp/Glu/Tyr, Trt for Asn/Gln/His, Boc for Lys/Trp, and Pbf for Arg. Following coupling and Fmoc deprotection of the final AA, the resin was directly treated with TFA:TIS:H_2O (95:2.5:2.5, 10 mL) for 90 min. The reaction mixture was added to cold MTBE:hexanes (1:1) and the resulting precipitate was centrifuged at 4500 rpm for 5 min, washed once more with MTBE:hexanes (1:1) and centrifuged at 4500 rpm for 5 min. The crude peptides were lyophilized from ^{t}BuOH:H_2O (1:1) and purified with reverse phase HPLC. Pure fractions were pooled and lyophilized to yield the desired linear peptide products in >95% purity as white powders.

Synthesis of C-terminal amide peptides.

Rink Amide resin (150 mg, 0.684 mmol.g^{-1}) was loaded into the CEM Liberty Blue peptide synthesizer for a 0.1mmol scale. Linear peptide encompassing the first amino acid to the last amino acid were assembled using microwave irradiation (resin bound AA:Fmoc-AA:DIC:Oxyma, 1:5:10:5 molar eq.). DMF was used as solvent and Fmoc deprotections were carried out with piperidine:DMF (1:4, v:v). Amino acid side chains were protected as follows: ^{t}Bu for Ser/Asp/Glu/Tyr, Trt for Asn/Gln/His, Boc for Lys/Trp, and Pbf for Arg. Following coupling and Fmoc deprotection of the final AA, the resin was directly treated with TFA:TIS:H_2O (95:2.5:2.5, 10 mL) for 90 min. The reaction mixture was added to cold MTBE:hexanes (1:1) and the resulting precipitate was centrifuged at 4500 rpm for 5 min, washed once more with MTBE:hexanes (1:1) and centrifuged at 4500 rpm for 5 min. The crude peptides were lyophilized from ^{t}BuOH:H_2O (1:1) and purified with reverse phase HPLC. Pure fractions were pooled and lyophilized to yield the desired linear peptide products in >95% purity as white powders.

Biological data of peptides 1-22 and PMBN

Checkerboard assays and FICi data against *E.coli* BW25113 with erythromycin

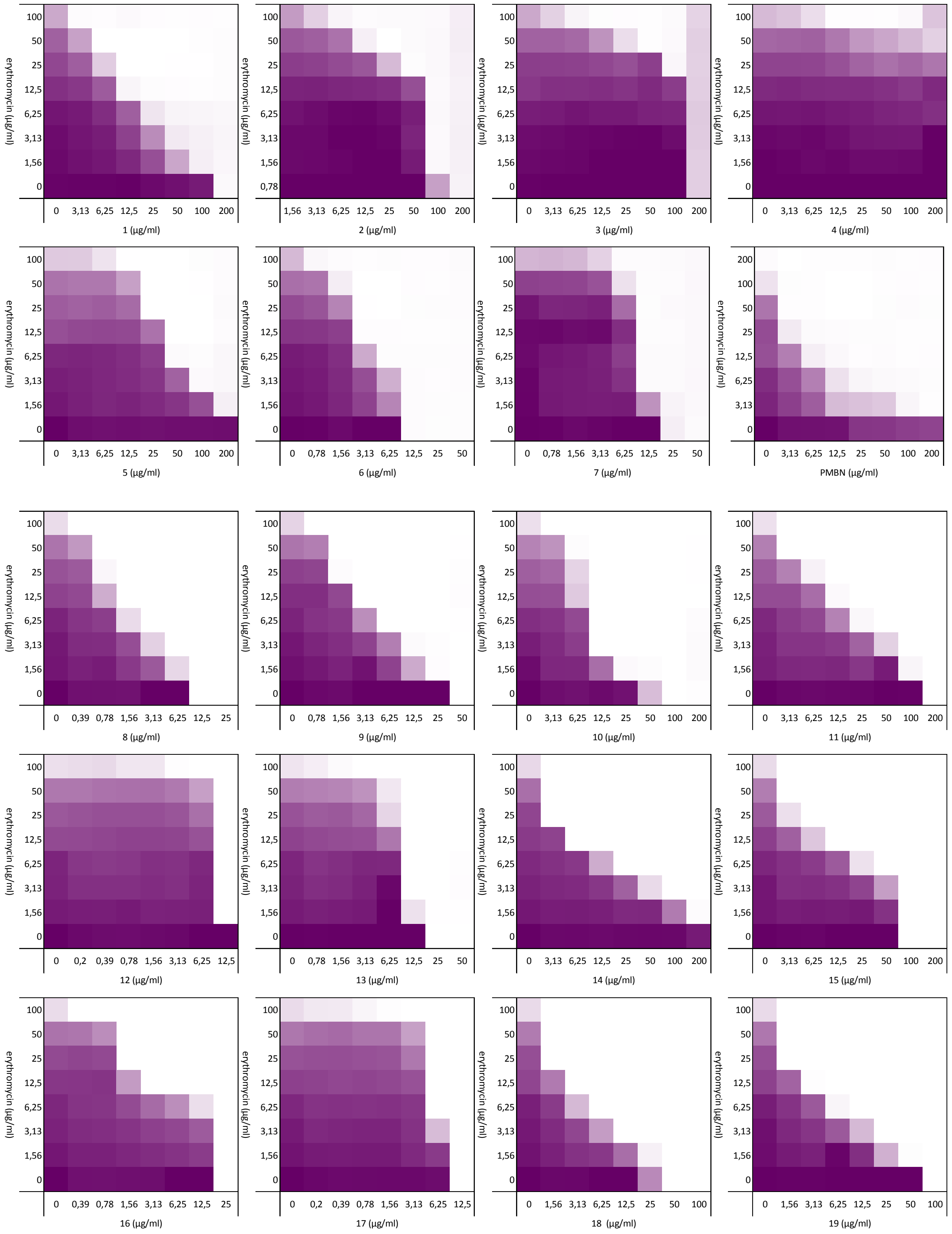

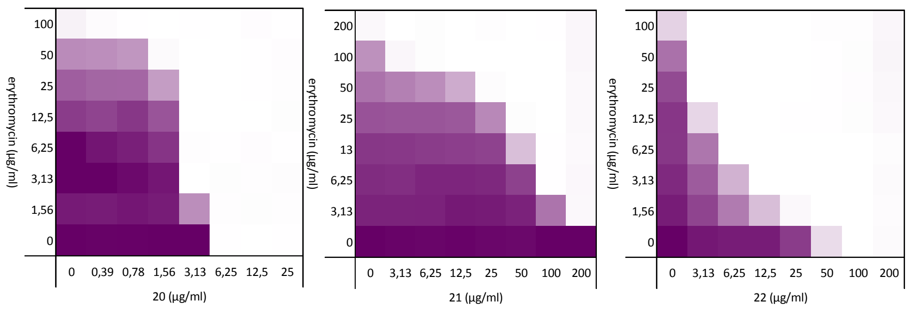

Figure S1 Checkerboard assays of the peptides **1-22** and PMBN in combination with erythromycin versus *E.coli* BW25113. OD_{600} values were measured using a plate reader and transformed to a gradient: purple represents growth, white represents no growth.

Table S1 Synergetic data of peptides **1-22** and PMBN of the checkerboard assays with erythromycin in **Figure S1**.

	MIC_{pep}	MSC_{pep}	MIC_{ery}	MSC_{ery}	FICi
**1**	200	25	>100	12.5	0.1875
**2**	200	50	>100	25	0.3750
**3**	200	50	>100	50	0.5000
**4**	>200	12.5	>100	100	>0.5 [1]
**5**	>200	50	>100	6.25	0.1563
**6**	12.5	3.125	>100	12.5	0.3125
**7**	50	12.5	>100	3.125	0.2656
**8**	12.5	1.563	>100	12.5	0.1875
**9**	50	3.125	>100	12.5	0.1250
**10**	100	12.5	>100	3.125	0.1406
**11**	200	25	>100	12.5	0.1875
**12**	25	12.5	>100	1.563	>0.5 [1]
**13**	25	12.5	>100	3.125	>0.5 [1]
**14**	>200	6.25	>100	12.5	0.0781
**15**	100	12.5	>100	12.5	0.1875
**16**	25	3.125	>100	12.5	0.1875
**17**	12.5	6.25	>100	6.25	>0.5 [1]
**18**	50	3.125	>100	12.5	0.1250
**19**	100	3.125	>100	12.5	0.0938
**20**	6.25	3.13	>100	3.125	>0.5 [1]
**21**	>200	50	200	25	0.2500
**22**	100	6.25	>100	6.25	0.0938
**PMBN**	>200	25	200	12.5	0.1250

[1] Synergy is defined as FICi ≤ 0.5 [1].

Checkerboard assays and FICi data against *E.coli* BW25113 with rifampicin

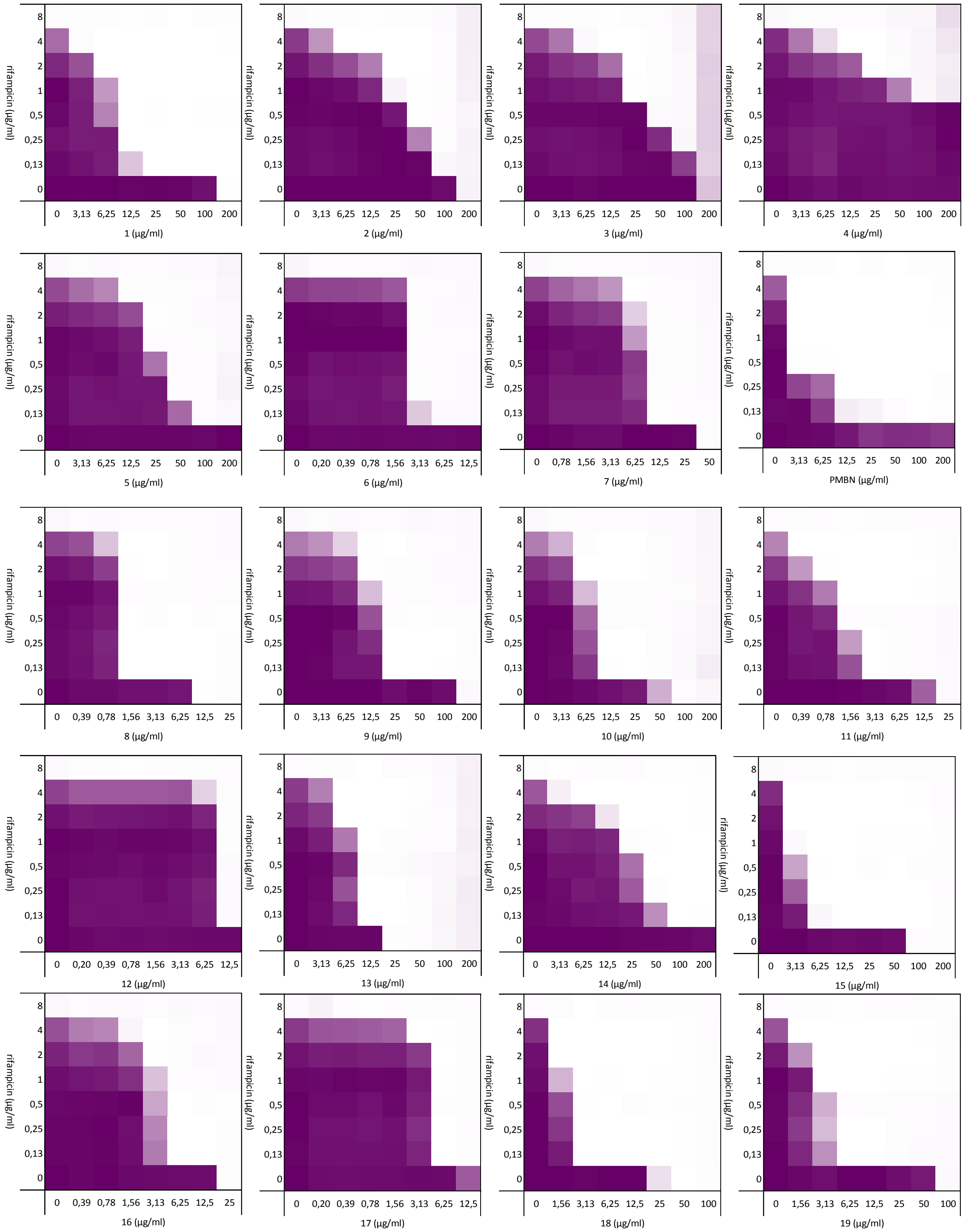

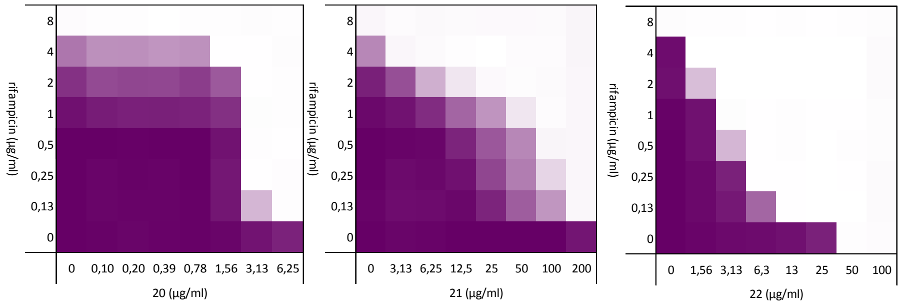

Figure S2 Checkerboard assays of the peptides **1-22** and PMBN in combination with rifampicin versus *E.coli* BW25113. OD_{600} values were measured using a plate reader and transformed to a gradient: purple represents growth, white represents no growth.

Table S2 Synergetic data of peptides **1-22** and PMBN of the checkerboard assays with rifampicin in **Figure S2**.

	MIC_{pep}	MSC_{pep}	MIC_{rif}	MSC_{rif}	FICi
**1**	200	12.5	8	0.25	0.0938
**2**	200	50	8	0.5	0.3125
**3**	>200	25	8	1	0.1875
**4**	>200	25	8	2	0.3125
**5**	>200	50	8	0.25	0.1563
**6**	25	3.125	8	0.25	0.1563
**7**	50	12.5	8	0.125	0.2656
**8**	12.5	1.563	8	0.125	0.1406
**9**	200	25	8	0.125	0.1406
**10**	100	12.5	8	0.125	0.1406
**11**	25	1.563	8	0.25	0.1250
**12**	25	12.5	8	0.125	>0.5 [1]
**13**	25	6.25	8	2	0.5000
**14**	>200	50	8	0.125	0.1406
**15**	100	6.25	8	0.25	0.0938
**16**	25	6.25	8	0.125	0.2656
**17**	25	6.25	8	0.125	0.2656
**18**	50	3.125	8	0.125	0.0781
**19**	100	6.25	8	0.125	0.0781
**20**	12.5	3.125	8	0.25	0.2813
**21**	>200	50	8	2	0.3750
**22**	50	6.25	8	0.25	0.1406
**PMBN**	>200	12.5	8	0.25	0.0625

[1] Synergy is defined as FICi ≤ 0.5 [1].

Hemolysis assay of peptides 1-22

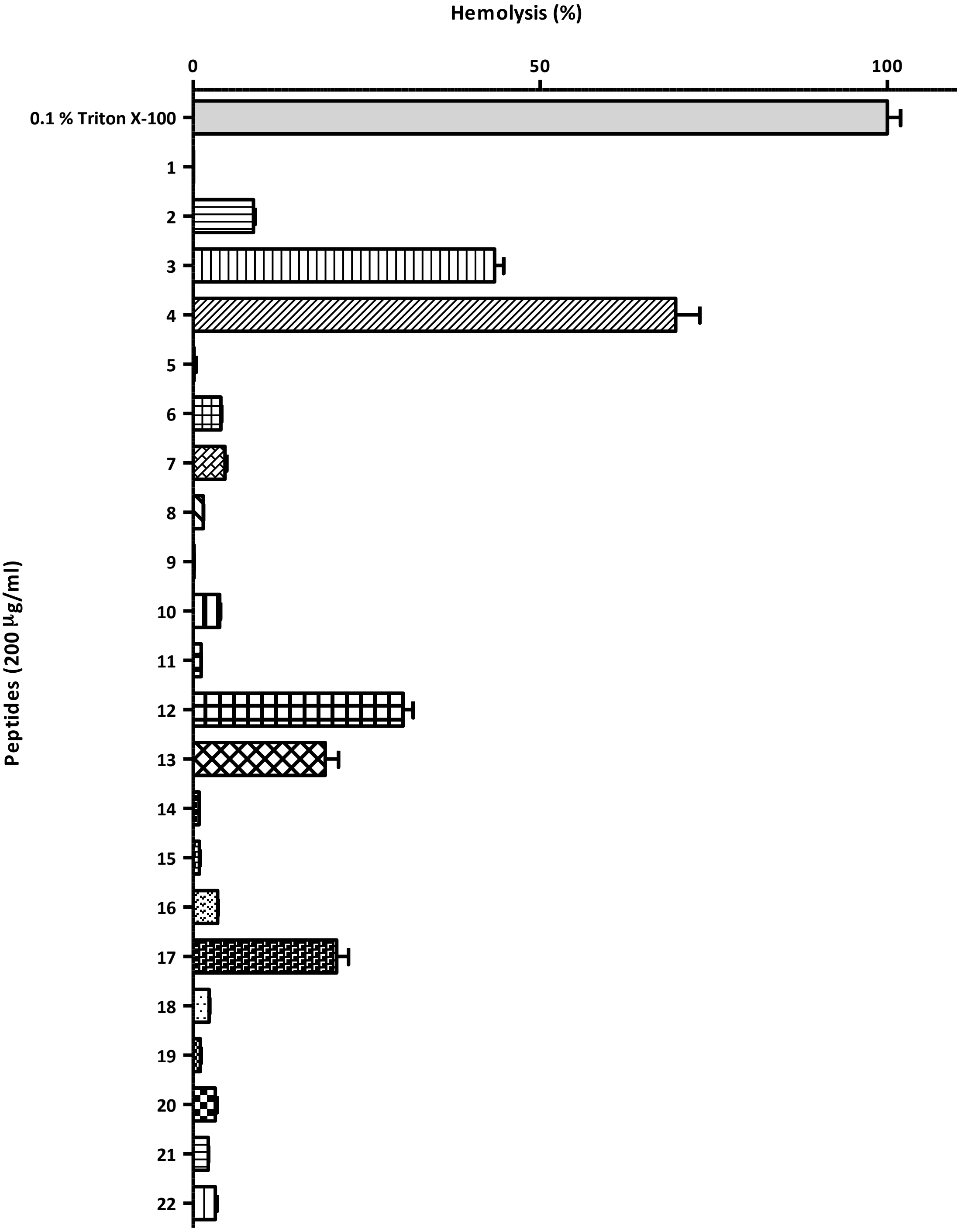

Figure S3 Hemolytic activity of peptides **1-22** (200 µg/ml). The hemolysis assay was performed as described in materials and methods. Values above 10% were defined as hemolytic for the peptides **1-4** in a previous study [2]. Error bars represent the standard deviation based on n=3 technical replicates.

Table S3 Hemolytic activity of peptides **1-22** (200 µg/ml). The hemolysis assay was performed as described in materials and methods. Values above 10% were defined as hemolytic for the peptides **1-4** in a previous study [2].

Compound	**Peptide sequence**	**Hemolysis (%)**
1	H_2N-VFRLKKWIQKVI-COOH	0.1
2	H_2N-HVFRLKKWIQKVIDQFGE-COOH	8.7
3	H_2N-FYTHVFRLKKWIQKVIDQFGE-COOH	43.4
4	H_2N-GKYGFYTHVFRLKKWIQKVIDQFGE-COOH	69.5
5	Ac-VFRLKKWIQKVI-COOH	0.2
6	H_2N-VFRLKKWIQKVI-$CONH_2$	4.0
7	Ac-VFRLKKWIQKVI-$CONH_2$	4.6
8	H_2N-**A**FRLKKWIQKVI-$CONH_2$	1.5
9	H_2N-V**A**RLKKWIQKVI-$CONH_2$	0.1
10	H_2N-VF**A**LKKWIQKVI-$CONH_2$	3.8
11	H_2N-VFR**A**KKWIQKVI-$CONH_2$	1.1
12	H_2N-VFRL**A**KWIQKVI-$CONH_2$	30.2
13	H_2N-VFRLK**A**WIQKVI-$CONH_2$	19.0
14	H_2N-VFRLKK**A**IQKVI-$CONH_2$	0.9
15	H_2N-VFRLKKW**A**QKVI-$CONH_2$	0.9
16	H_2N-VFRLKKWI**A**KVI-$CONH_2$	3.5
17	H_2N-VFRLKKWIQ**A**VI-$CONH_2$	20.7
18	H_2N-VFRLKKWIQK**A**I-$CONH_2$	2.3
19	H_2N-VFRLKKWIQKV**A**-$CONH_2$	1.0
20	H_2N-vfrlkkwiqkvi-$CONH_2$	3.2
21	H_2N-IVKQIWKKLRFV-$CONH_2$	2.2
22	H_2N-ivkqiwkklrfv-$CONH_2$	3.2

LPS competition assay of peptide 6

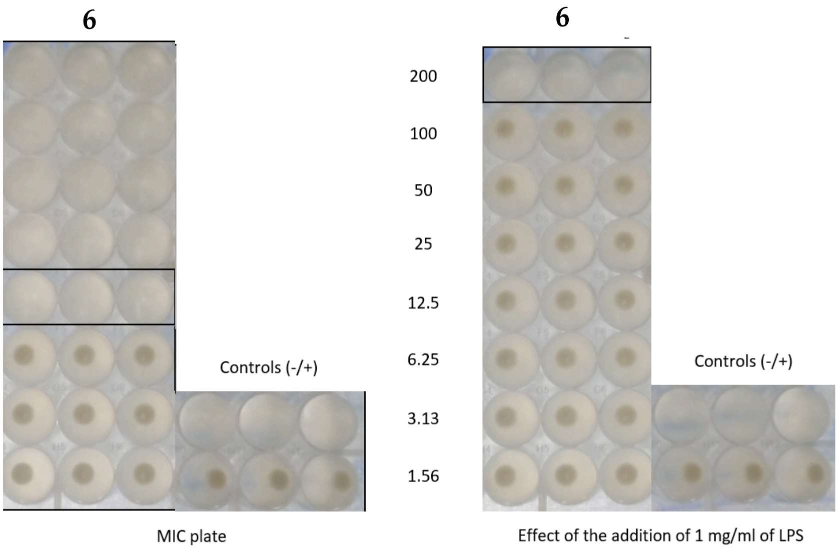

Figure S4 LPS competition assay of **6** with *E.coli* BW25113 in LB as described in materials and methods. A visual read-out was performed after centrifuging the plates for 2 minutes at 3000 rpm.

Table S4 Overview of LPS competition results using LB as medium. All results are obtained against *E.coli* BW25113 as shown in **Figure S4**.

	Peptide sequence	MIC	+ 1.0 mg/ml LPS
**6**	H_2N-VFRLKKWIQKVI-$CONH_2$	12.5	200

Synergy data of peptides 6, 10, 14, 19, and PMBN

Checkerboard assays and FICi data against *E.coli* BW25113 with novobiocin

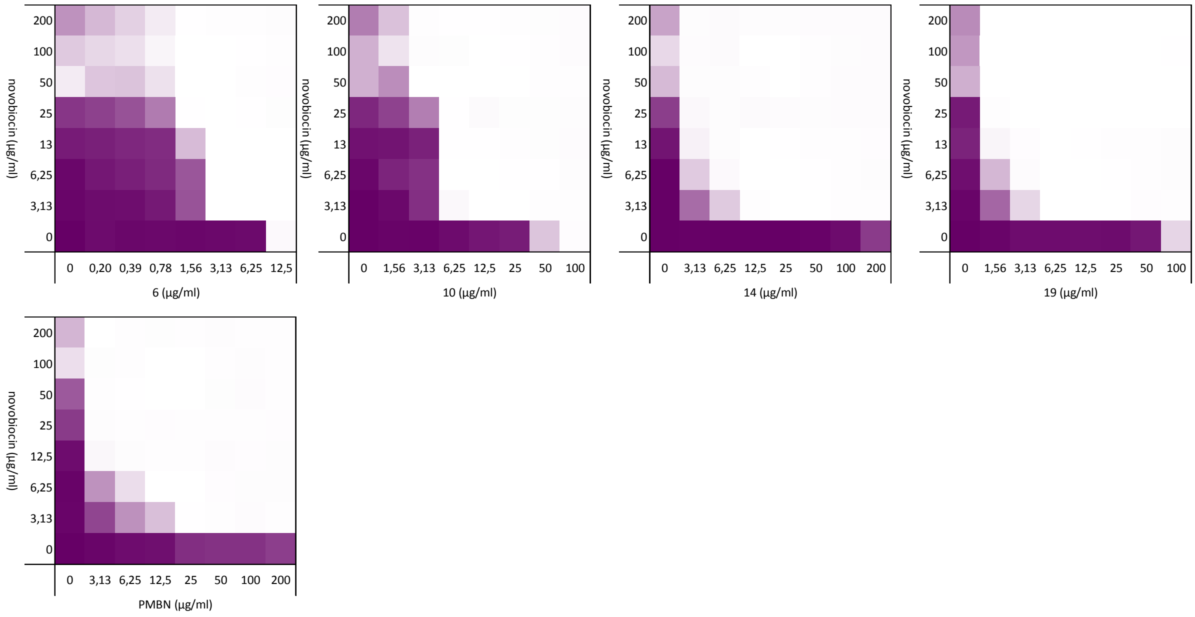

Figure S5 Checkerboard assays of the peptides **6**, **10**, **14**, **19** and PMBN in combination with novobiocin versus *E.coli* BW25113. OD_{600} values were measured using a plate reader and transformed to a gradient: purple represents growth, white represents no growth.

Table S5 Synergetic data of peptides **6**, **10**, **14**, **19** and PMBN of the checkerboard results for *E.coli* BW25113 with novobiocin displayed in **Figure S5**.

	$MIC_{peptide}$	$MSC_{peptide}$	MIC_{novo}	MSC_{novo}	FICi
**6**	12.5	1.563	>200	25	0.1875
**10**	100	6.25	>200	6.25	0.0781
**14**	>200	6.25	>200	6.25	0.0313
**19**	200	6.25	>200	3.125	0.0390
**PMBN**	>200	12.5	>200	6.25	0.0469

Checkerboard assays and FICi data against *E.coli* BW25113 with vancomycin

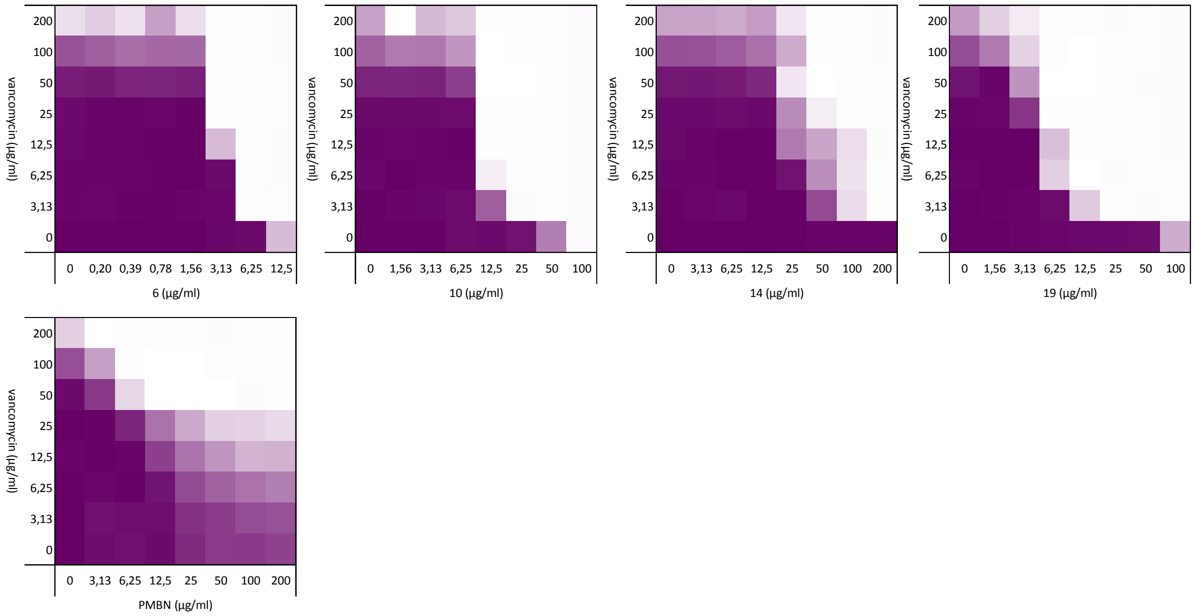

Figure S6 Checkerboard assays of the peptides **6**, **10**, **14**, **19** and PMBN in combination with vancomycin versus *E.coli* BW25113. OD_{600} values were measured using a plate reader and transformed to a gradient: purple represents growth, white represents no growth.

Table S6 Synergetic data of peptides **6**, **10**, **14**, **19** and PMBN of the checkerboard results for *E.coli* BW25113 with vancomycin displayed in **Figure S6**.

	$MIC_{peptide}$	$MSC_{peptide}$	MIC_{vanco}	MSC_{vanco}	FICi
**6**	25	3.125	>200	25	0.1875
**10**	100	12.5	>200	12.5	0.1563
**14**	>200	50	>200	50	0.2500
**19**	200	12.5	>200	6.25	0.0781
**PMBN**	>200	12.5	>200	50	0.1563

Synergy data of peptides 6, 10, 14, and 19 with rifampicin

E. coli ATCC25922

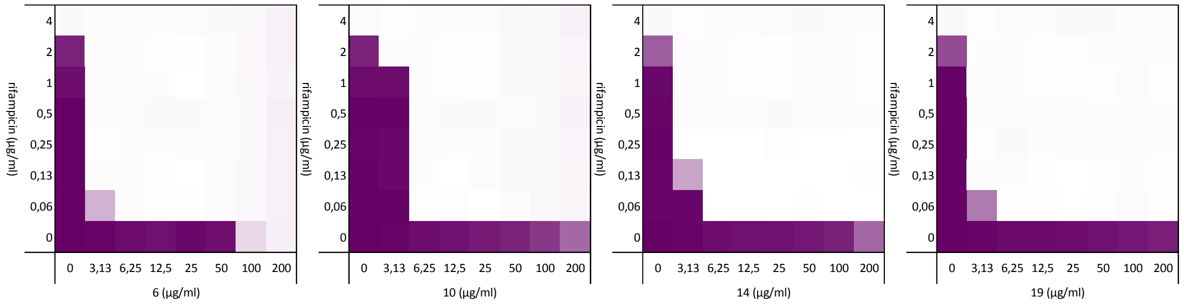

Figure S7 Checkerboard assays of the peptides **6**, **10**, **14**, and **19** in combination with rifampicin versus *E.coli* ATCC25922. OD_{600} values were measured using a plate reader and transformed to a gradient: purple represents growth, white represents no growth.

Table S7 Synergistic data of peptides **6**, **10**, **14**, and **19** of the checkerboard results for *E.coli* ATCC25922 with rifampicin displayed in **Figure S7**.

	$MIC_{peptide}$	$MSC_{peptide}$	MIC_{rif}	MSC_{rif}	FICi
**6**	200	6.25	4	0.063	0.0469
**10**	>200	6.25	4	0.063	0.0313
**14**	>200	6.25	4	0.063	0.0313
**19**	>200	6.25	4	0.063	0.0313

E. coli W3110

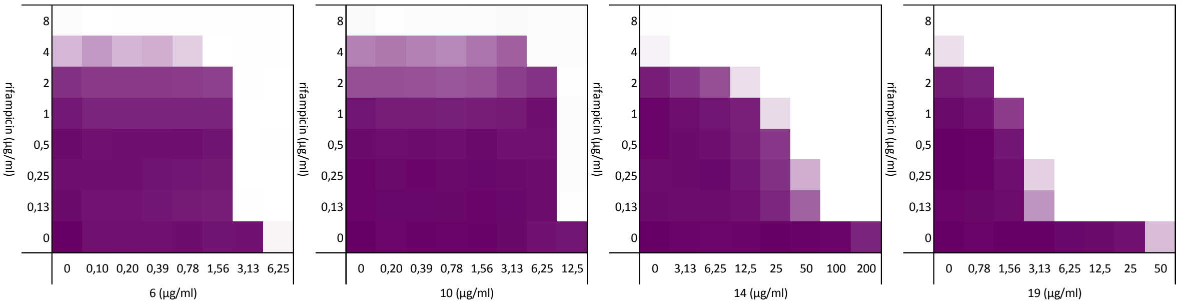

Figure S8 Checkerboard assays of the peptides **6**, **10**, **14**, and **19** in combination with rifampicin versus *E.coli* W3110. OD_{600} values were measured using a plate reader and transformed to a gradient: purple represents growth, white represents no growth.

Table S8 Synergistic data of peptides **6**, **10**, **14**, and **19** of the checkerboard results for *E.coli* W3110 with rifampicin displayed in **Figure S8**.

	$MIC_{peptide}$	$MSC_{peptide}$	MIC_{rif}	MSC_{rif}	FICi
**6**	6.25	3.125	8	0.125	>0.5 [1]
**10**	25	12.5	8	0.125	>0.5 [1]
**14**	>200	50	8	0.5	0.1875
**19**	100	3.125	8	0.5	0.0782

[1] Synergy is defined as FICi ≤ 0.5 [1].

E. coli mcr-1

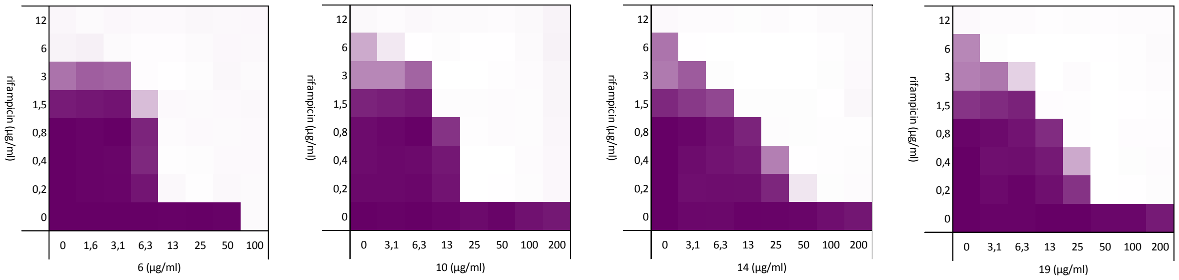

Figure S9 Checkerboard assays of the peptides **6**, **10**, **14**, and **19** in combination with rifampicin versus *E.coli* mcr-1. OD_{600} values were measured using a plate reader and transformed to a gradient: purple represents growth, white represents no growth.

Table S9 Synergistic data of peptides **6**, **10**, **14**, and **19** of the checkerboard results for *E.coli* mcr-1 with rifampicin displayed in **Figure S9**.

	**MIC$_{peptide}$**	**MSC$_{peptide}$**	**MIC$_{rif}$**	**MSC$_{rif}$**	**FICi**
**6**	100	12.5	12	0.188	0.1406
**10**	>200	25	12	0.188	0.0781
**14**	>200	25	12	0.75	0.1250
**19**	>200	25	12	0.75	0.1250

E. coli EQASmcr-1/EQAS 2016 412016126

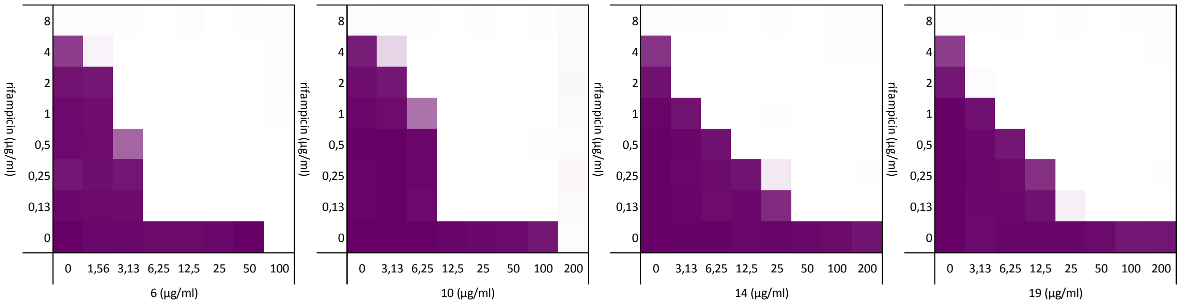

Figure S10 Checkerboard assays of the peptides **6**, **10**, **14**, and **19** in combination with rifampicin versus *E.coli* EQASmcr-1/EQAS 2016 412016126. OD_{600} values were measured using a plate reader and transformed to a gradient: purple represents growth, white represents no growth.

Table S10 Synergistic data of peptides **6**, **10**, **14**, and **19** of the checkerboard results for *E.coli* EQASmcr-1/EQAS 2016 412016126 with rifampicin displayed in **Figure S10**.

	$MIC_{peptide}$	$MSC_{peptide}$	MIC_{rif}	MSC_{rif}	FICi
**6**	100	6.25	8	0.125	0.0781
**10**	200	12.5	8	0.125	0.0781
**14**	>200	12.5	8	0.5	0.0938
**19**	>200	12.5	8	0.5	0.0938

E. coli EQASmcr-2/EQAS 2016 KP37

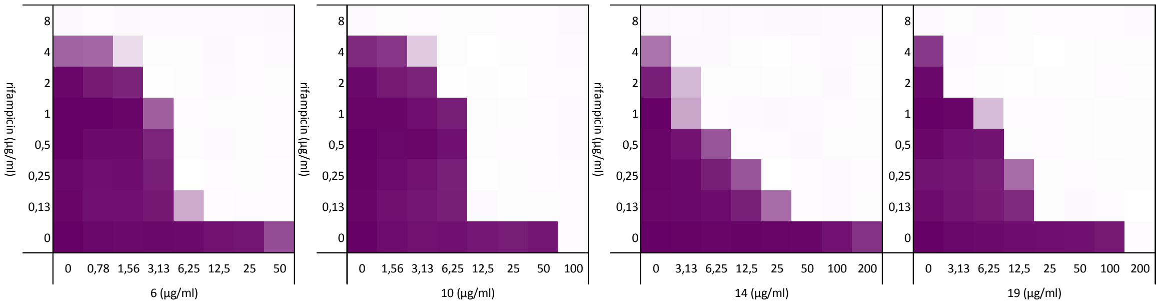

Figure S11 Checkerboard assays of the peptides **6**, **10**, **14**, and **19** in combination with rifampicin versus *E.coli* EQASmcr-2/EQAS 2016 KP37. OD_{600} values were measured using a plate reader and transformed to a gradient: purple represents growth, white represents no growth.

Table S11 Synergistic data of peptides **6**, **10**, **14**, and **19** of the checkerboard results for *E.coli* EQASmcr-2/EQAS 2016 KP37 with rifampicin displayed in **Figure S11**.

	$MIC_{peptide}$	$MSC_{peptide}$	MIC_{rif}	MSC_{rif}	FICi
**6**	100	6.25	8	0.25	0.0938
**10**	100	12.5	8	0.125	0.1406
**14**	>200	25	8	0.25	0.0938
**19**	200	12.5	8	0.5	0.1250

E. coli EQASmcr-3/EQAS 2017 2013-SQ352

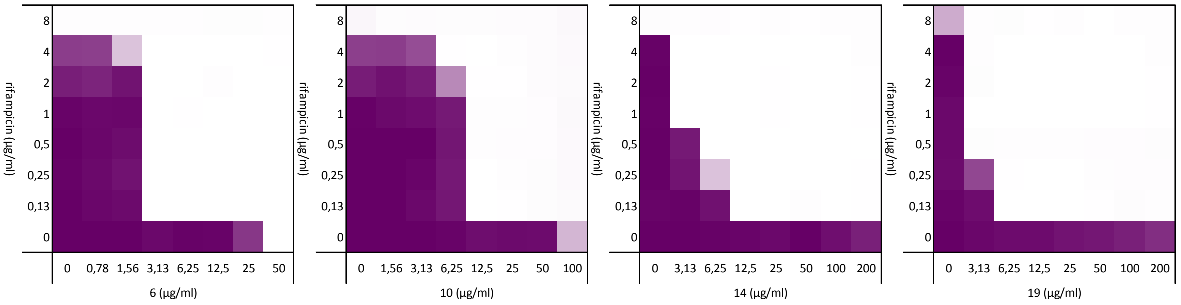

Figure S12 Checkerboard assays of the peptides **6**, **10**, **14**, and **19** in combination with rifampicin versus *E.coli* EQASmcr-3/EQAS 2017 2013-SQ352. OD_{600} values were measured using a plate reader and transformed to a gradient: purple represents growth, white represents no growth.

Table S12 Synergistic data of peptides **6**, **10**, **14**, and **19** of the checkerboard results for *E.coli* EQASmcr-3/EQAS 2017 2013-SQ352 with rifampicin displayed in **Figure S12**.

	$MIC_{peptide}$	$MSC_{peptide}$	MIC_{rif}	MSC_{rif}	FICi
**6**	50	3.125	8	0.125	0.0781
**10**	200	12.5	8	0.125	0.0781
**14**	>200	12.5	8	0.125	0.0469
**19**	>200	6.25	8	0.125	0.3125

A. baumannii ATCC17978

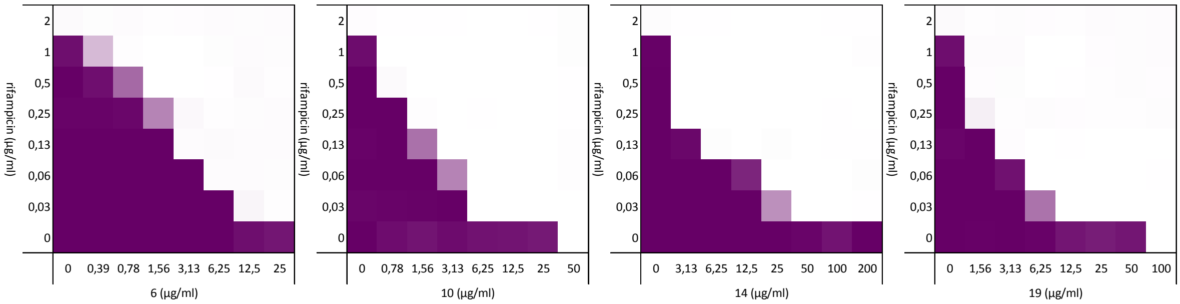

Figure S13 Checkerboard assays of the peptides **6**, **10**, **14**, and **19** in combination with rifampicin versus *A. baumannii* ATCC17978. OD_{600} values were measured using a plate reader and transformed to a gradient: purple represents growth, white represents no growth.

Table S13 Synergistic data of peptides **6**, **10**, **14**, and **19** of the checkerboard results for *A. baumannii* ATCC17978 with rifampicin displayed in **Figure S13**.

	$MIC_{peptide}$	$MSC_{peptide}$	MIC_{rif}	MSC_{rif}	FICi
**6**	50	3.125	2	0.125	0.1250
**10**	50	3.125	2	0.125	0.1250
**14**	>200	6.25	2	0.125	0.0781
**19**	100	6.25	2	0.063	0.0938

K. pneumoniae ATCC13883

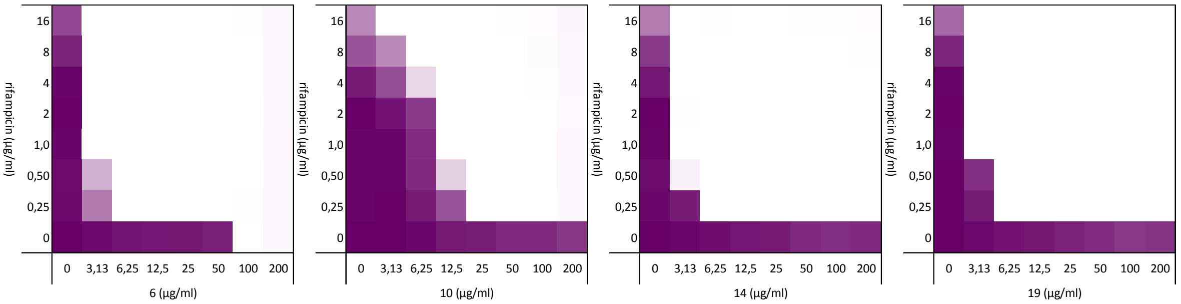

Figure S14 Checkerboard assays of the peptides **6**, **10**, **14**, and **19** in combination with rifampicin versus *K. pneumoniae* ATCC13883. OD_{600} values were measured using a plate reader and transformed to a gradient: purple represents growth, white represents no growth.

Table S14 Synergistic data of peptides **6**, **10**, **14**, and **19** of the checkerboard results for *K. pneumoniae* ATCC13883 with rifampicin displayed in **Figure S14**.

	$MIC_{peptide}$	$MSC_{peptide}$	MIC_{rif}	MSC_{rif}	FICi
**6**	100	3.125	32	1	0.0625
**10**	>200	12.5	32	1	0.0625
**14**	>200	6.25	32	0.25	0.0234
**19**	>200	6.25	32	0.25	0.0234

P. aeruginosa ATCC27853

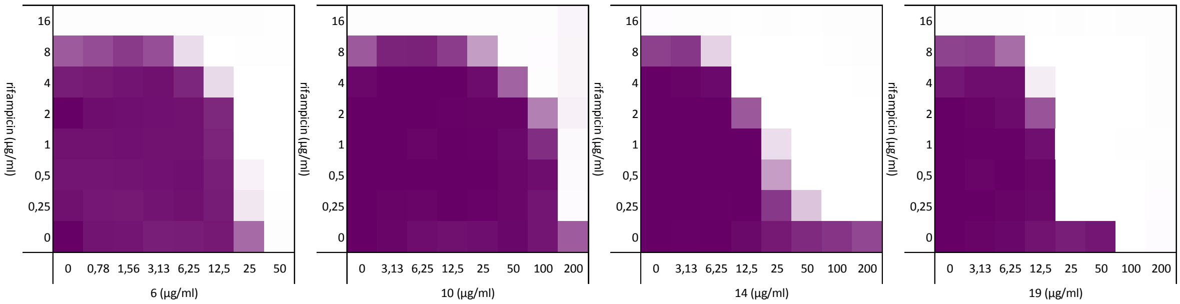

Figure S15 Checkerboard assays of the peptides **6**, **10**, **14**, and **19** in combination with rifampicin versus *P. aeruginosa* ATCC27853. OD_{600} values were measured using a plate reader and transformed to a gradient: purple represents growth, white represents no growth.

Table S15 Synergistic data of peptides **6**, **10**, **14**, and **19** of the checkerboard results for *P. aeruginosa* ATCC27853 with rifampicin displayed in **Figure S15**.

	$MIC_{peptide}$	$MSC_{peptide}$	MIC_{rif}	MSC_{rif}	FICi
**6**	50	25	16	1	>0.5 [1]
**10**	>200	100	16	4	0.2500
**14**	>200	50	16	0.5	0.1563
**19**	100	25	16	0.25	0.2656

[1] Synergy is defined as FICi ≤ 0.5 [1].

Membrane permeability assay using NPN

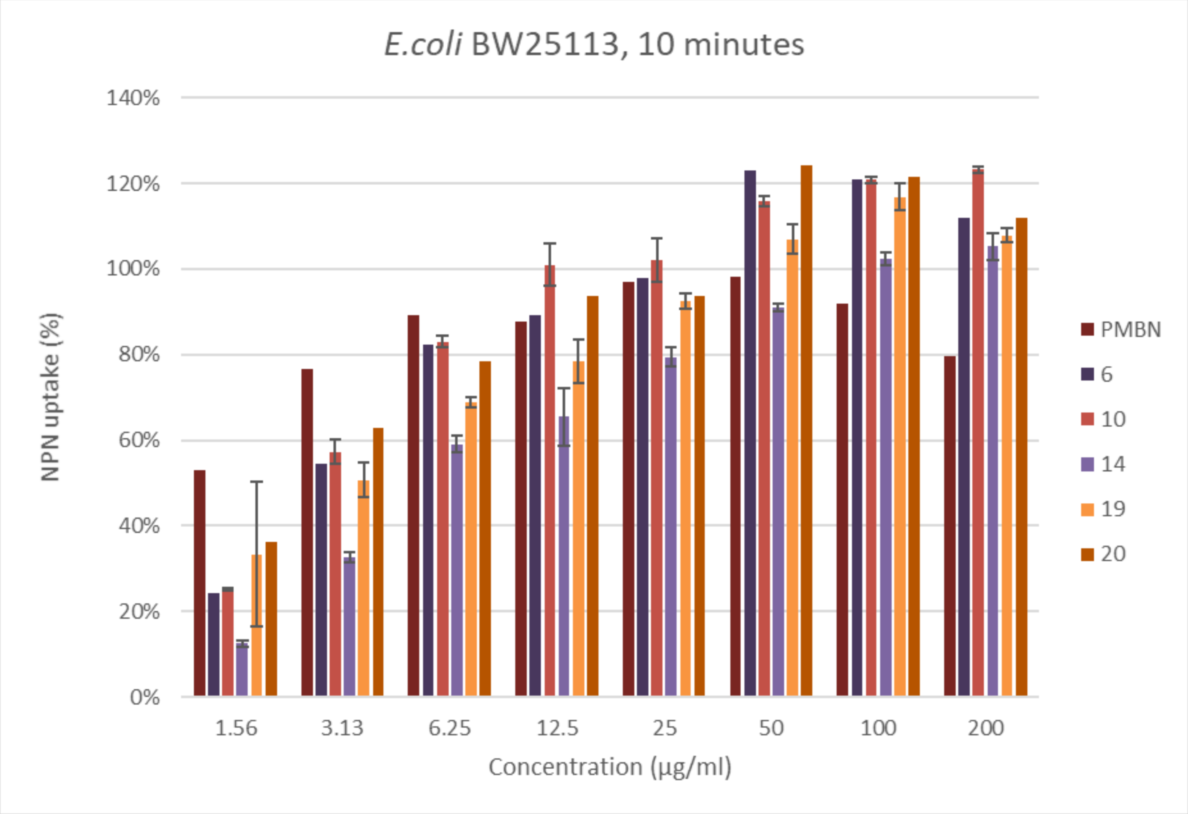

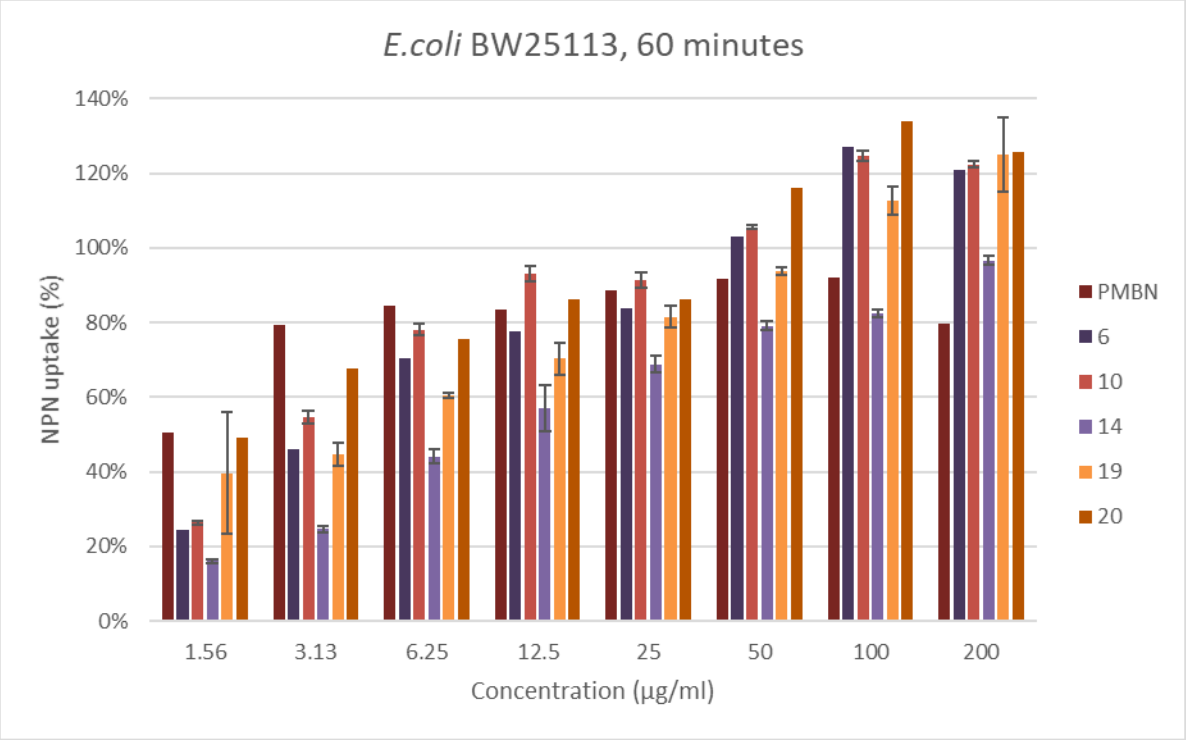

Figure S16 Permeabilization assay of *E.coli* BW25113 using *N*-Phenyl-1-naphthylamine as fluorescent probe. The read-out was performed using a platereader with λex 355 nm and λem 420 nm. The NPN uptake values shown are relative to the uptake signal obtained upon treating the cells with 100 µg/ml colistin as previously reported [3]. Error bars represent the standard deviation based on n=3 technical replicates.

Peptide characterization and analysis

HRMS characterization

Table S16 Overview of the HRMS results obtained using a Shimadzu Nexera X2 UHPLC system with a Waters Acquity HSS C18 column (2.1 × 100 mm, 1.8 µm) at 30 °C and equipped with a diode array detector. This system was connected to a Shimadzu 9030 QTOF mass spectrometer (ESI ionisation) calibrated internally with Agilent's API-TOF reference mass solution kit (5.0 mM purine, 100.0 mM ammonium trifluoroacetate and 2.5 mM hexakis(1H,1H,3H-tetrafluoropropoxy)phosphazine) diluted to achieve a mass count of 10000.

	Peptide sequence	[M+H+] calculated	[M+H+] found
**1**	H_2N-VFRLKKWIQKVI-COOH	1557.9998	1557.9993
**2**	H_2N-HVFRLKKWIQKVIDQFGE-COOH	2271.2767	2271.2791
**3**	H_2N-FYTHVFRLKKWIQKVIDQFGE-COOH	2682.4561	2682.4579
**4**	H_2N-GKYGFYTHVFRLKKWIQKVIDQFGE-COOH	3087.6573	1544.3326[1]
**5**	Ac-VFRLKKWIQKVI-COOH	1600.0104	1600.0110
**6**	H_2N-VFRLKKWIQKVI-$CONH_2$	1557.0158	1557.0153
**7**	Ac-VFRLKKWIQKVI-$CONH_2$	1599.0263	1599.0259
**8**	H_2N-**A**FRLKKWIQKVI-$CONH_2$	1528.9845	1528.9753
**9**	H_2N-V**A**RLKKWIQKVI-$CONH_2$	1480.9845	1480.9846
**10**	H_2N-VF**A**LKKWIQKVI-$CONH_2$	1471.9518	1471.9523
**11**	H_2N-VFR**A**KKWIQKVI-$CONH_2$	1514.9688	1514.9685
**12**	H_2N-VFRL**A**KWIQKVI-$CONH_2$	1499.9579	1499.9580
**13**	H_2N-VFRLK**A**WIQKVI-$CONH_2$	1499.9579	1499.9578
**14**	H_2N-VFRLKK**A**IQKVI-$CONH_2$	1441.9736	1441.9736
**15**	H_2N-VFRLKKW**A**QKVI-$CONH_2$	1514.9688	1514.9696
**16**	H_2N-VFRLKKWI**A**KVI-$CONH_2$	1499.9943	1500.0008
**17**	H_2N-VFRLKKWIQ**A**VI-$CONH_2$	1499.9579	1499.9646
**18**	H_2N-VFRLKKWIQK**A**I-$CONH_2$	1528.9845	1528.9912
**19**	H_2N-VFRLKKWIQKV**A**-$CONH_2$	1514.9688	1514.9753
**20**	H_2N-vfrlkkwiqkvi-$CONH_2$	1557.0158	1557.0156
**21**	H_2N-IVKQIWKKLRFV-$CONH_2$	1557.0158	1557.0151
**22**	H_2N-ivkqiwkklrfv-$CONH_2$	1557.0158	1557.0222

[1] In this case only the $[M+2H]^{2+}$ was observed

HPLC analysis

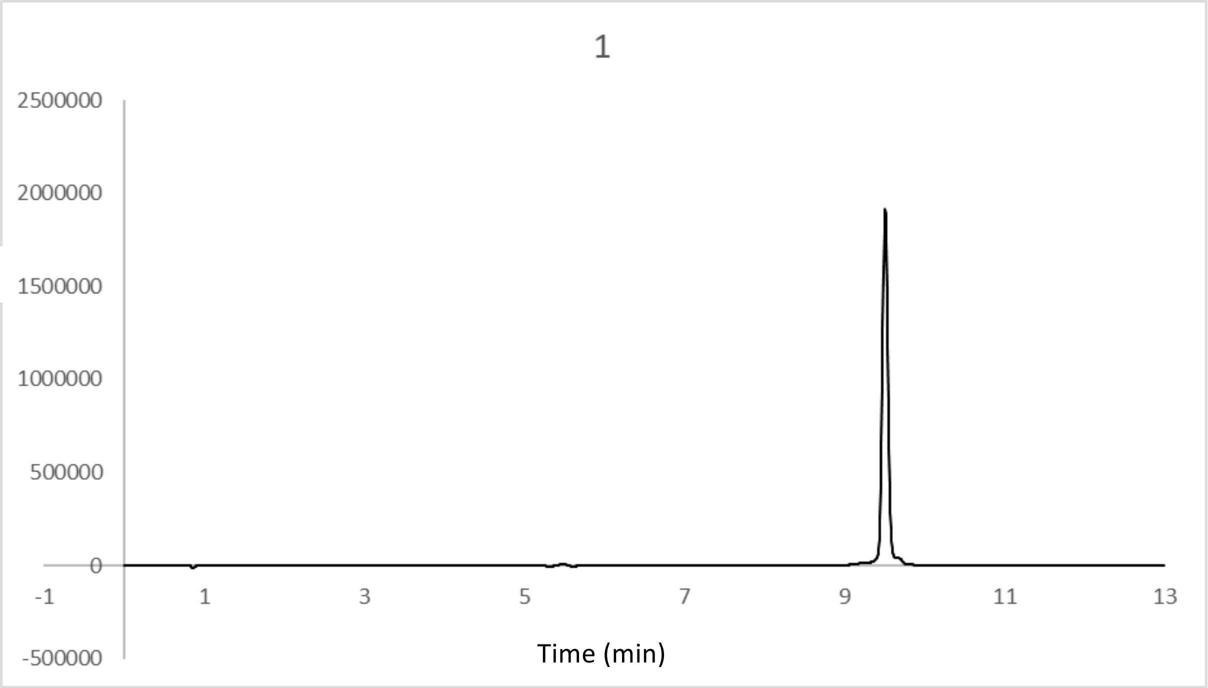

Compound 2 was run from a DMSO stock. 3min peak is DMSO

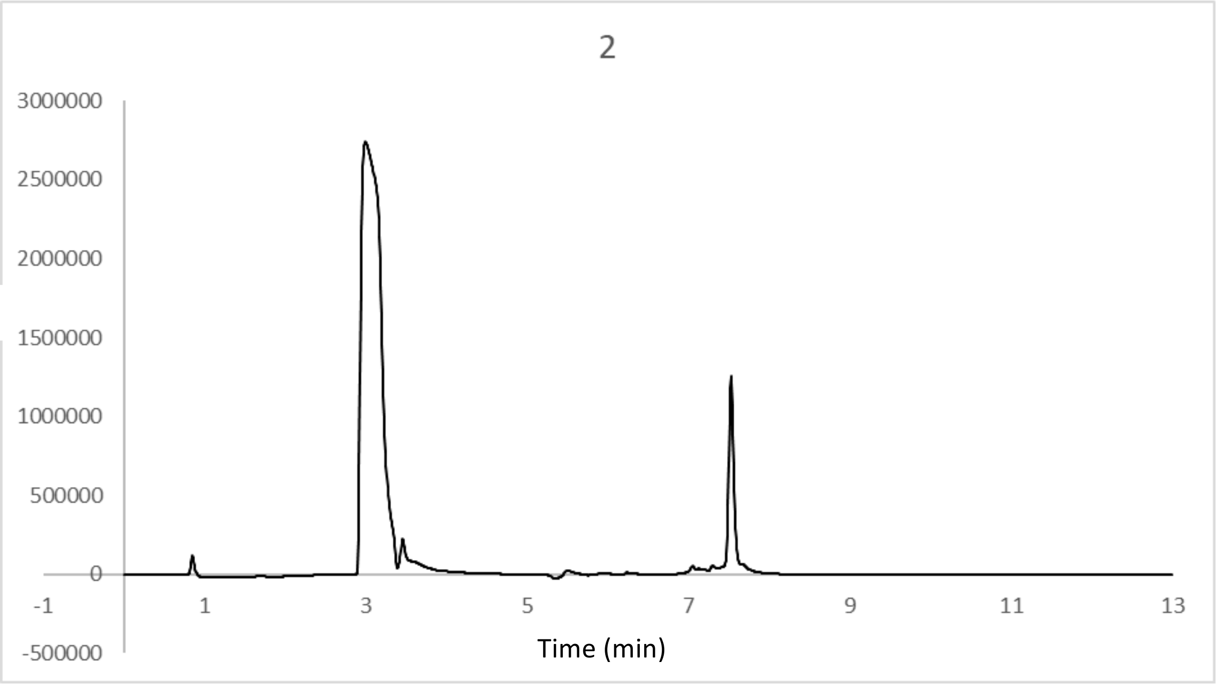

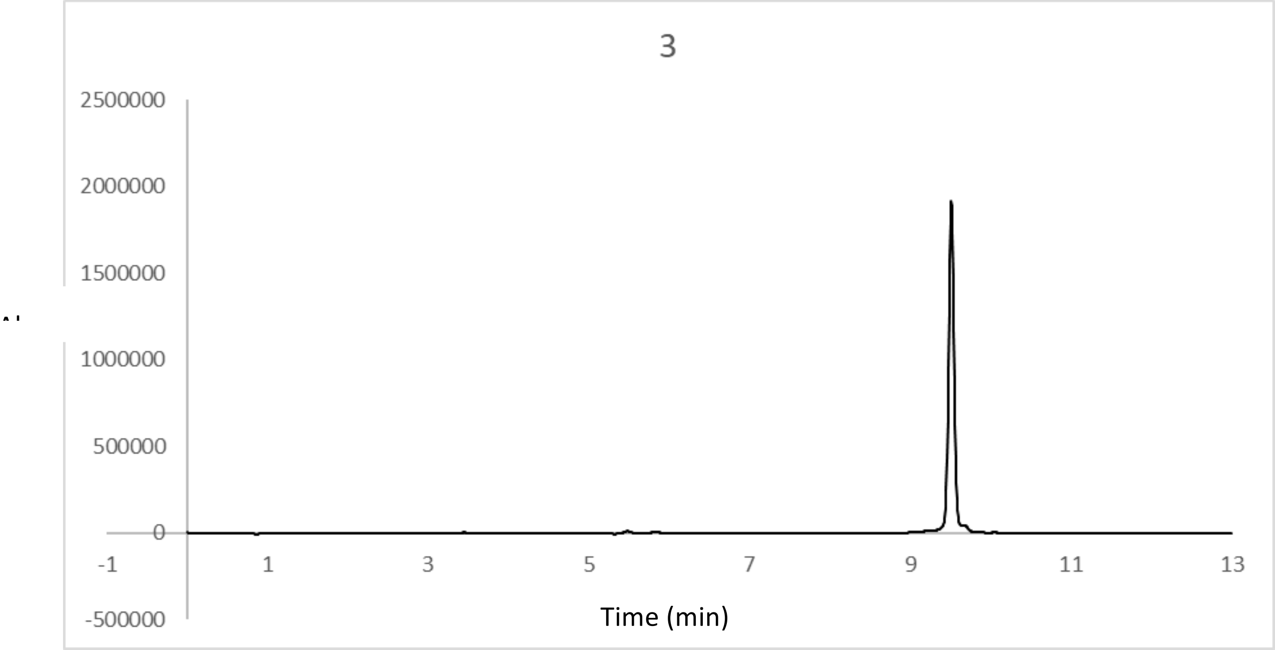
3
2500000
2000000
1500000
1000000
500000
0
-500000
-1
1
3
5
7
9
11
13
Time (min)

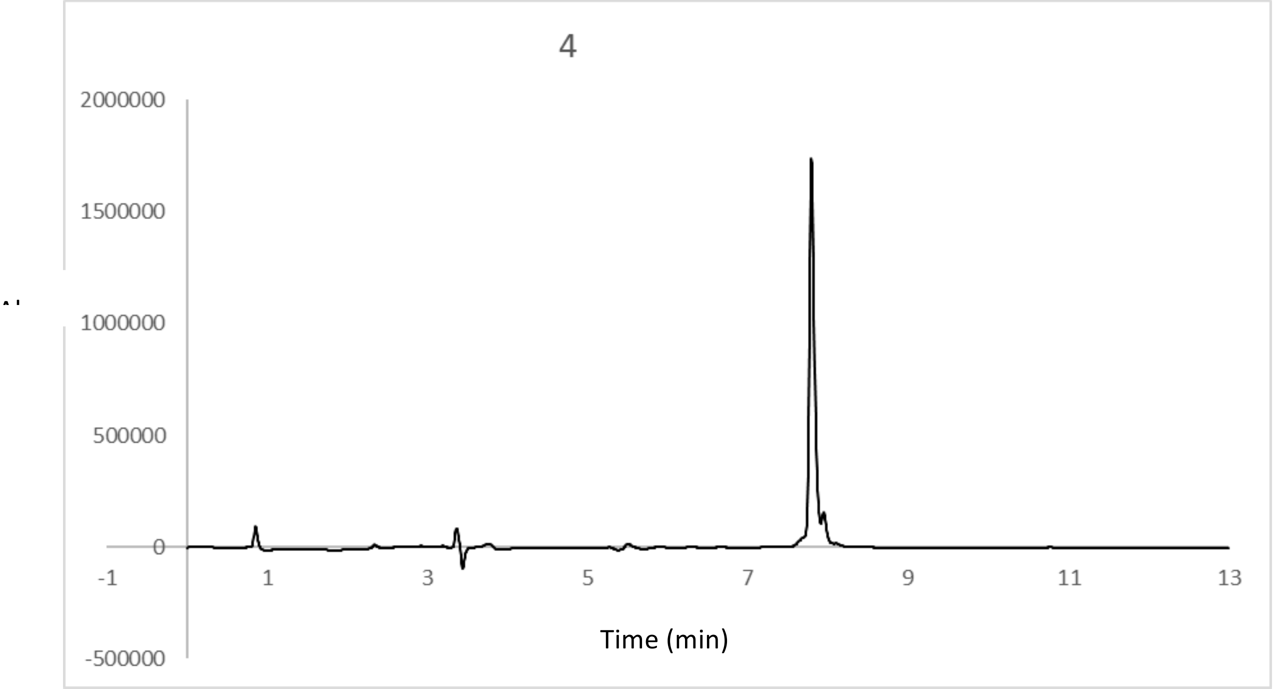
4
2000000
1500000
1000000
500000
0
-500000
-1
1
3
5
7
9
11
13
Time (min)

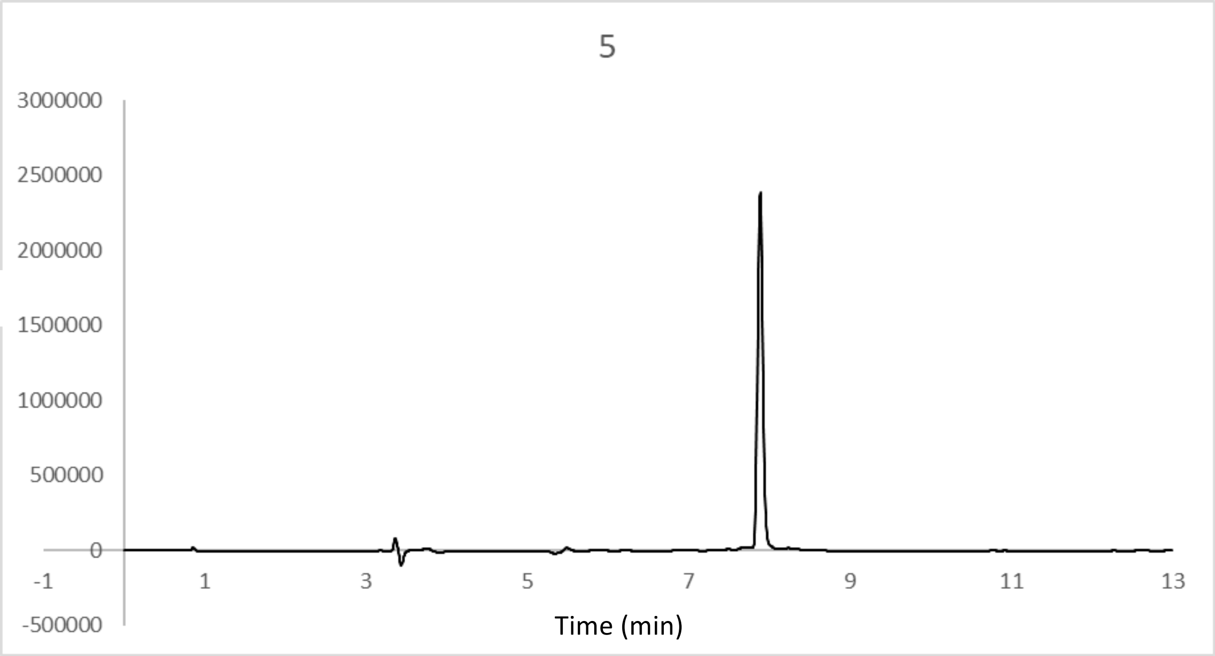
5
3000000
2500000
2000000
1500000
1000000
500000
0
-500000
-1
1
3
5
7
9
11
13
Time (min)

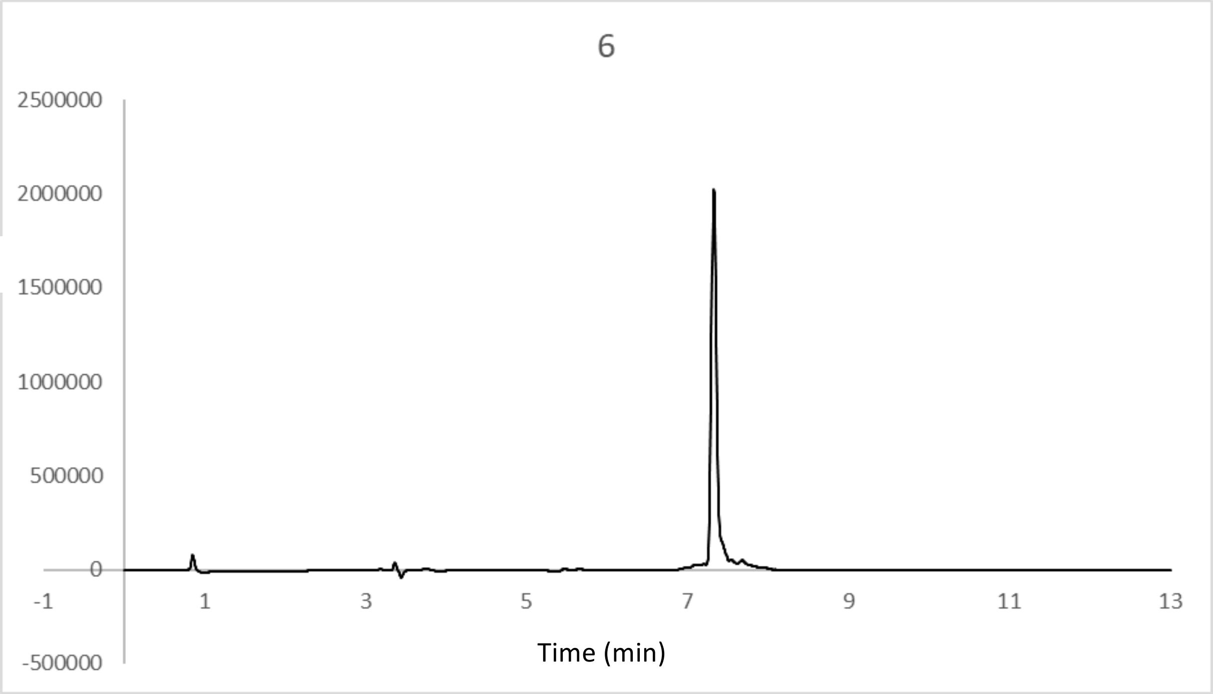
6
2500000
2000000
1500000
1000000
500000
0
-500000
-1
1
3
5
7
9
11
13
Time (min)

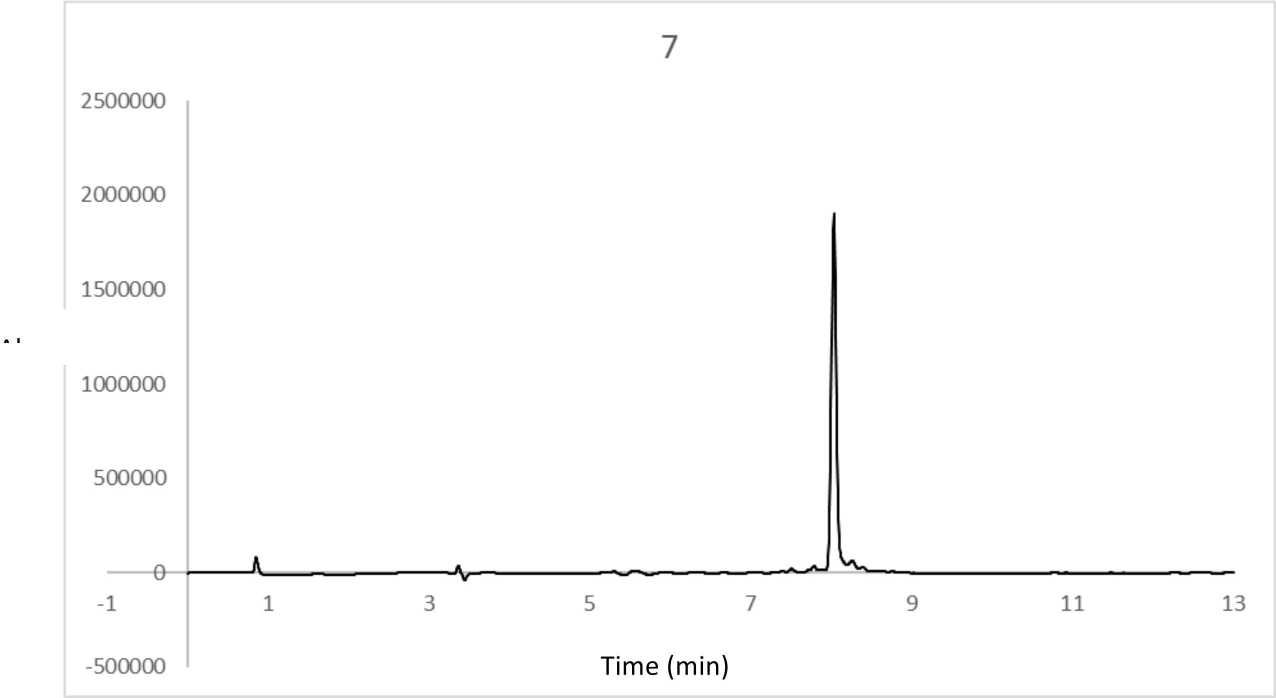
7
2500000
2000000
1500000
1000000
500000
0
-500000
-1
1
3
5
7
9
11
13
Time (min)

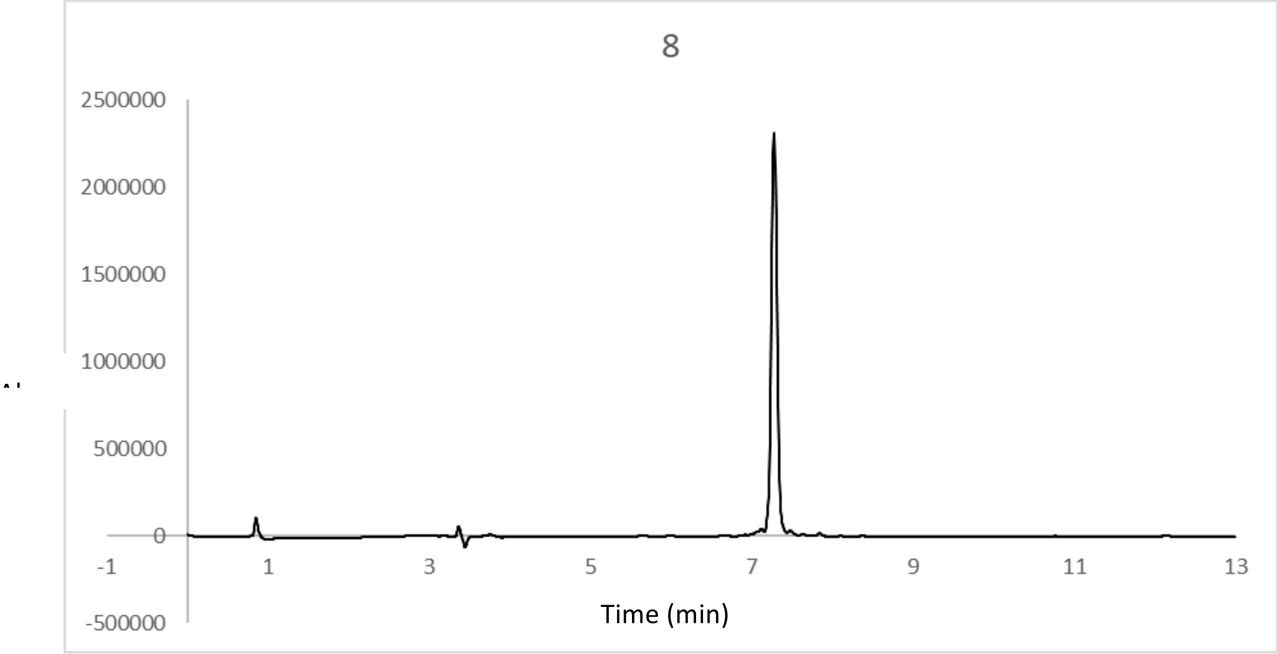
8
2500000
2000000
1500000
1000000
500000
0
-500000
-1
1
3
5
7
9
11
13
Time (min)

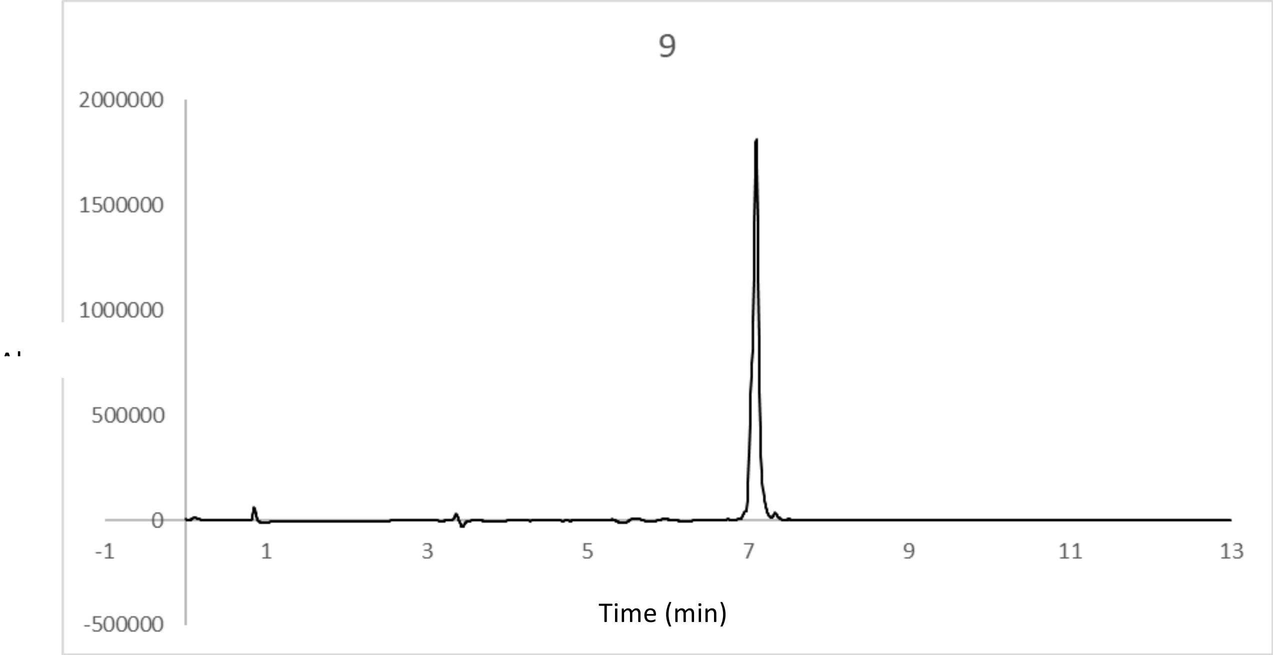
9
2000000
1500000
1000000
500000
0
-500000
-1
1
3
5
7
9
11
13
Time (min)

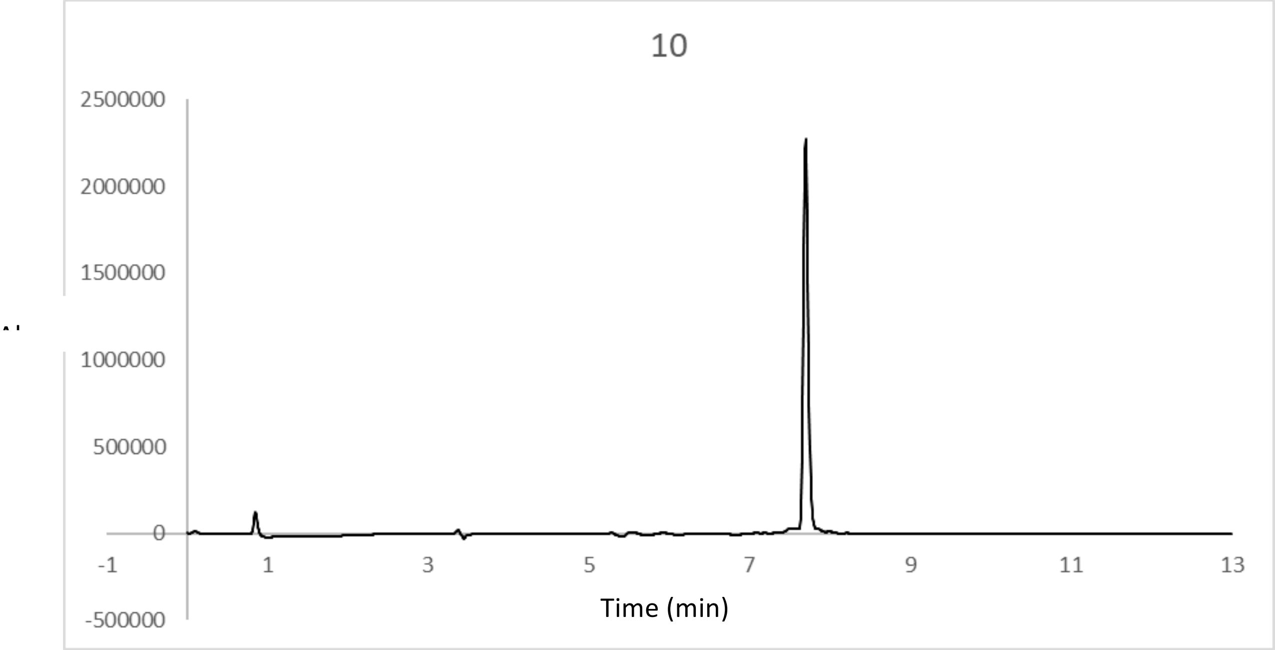
10
2500000
2000000
1500000
1000000
500000
0
-500000
-1
1
3
5
7
9
11
13
Time (min)

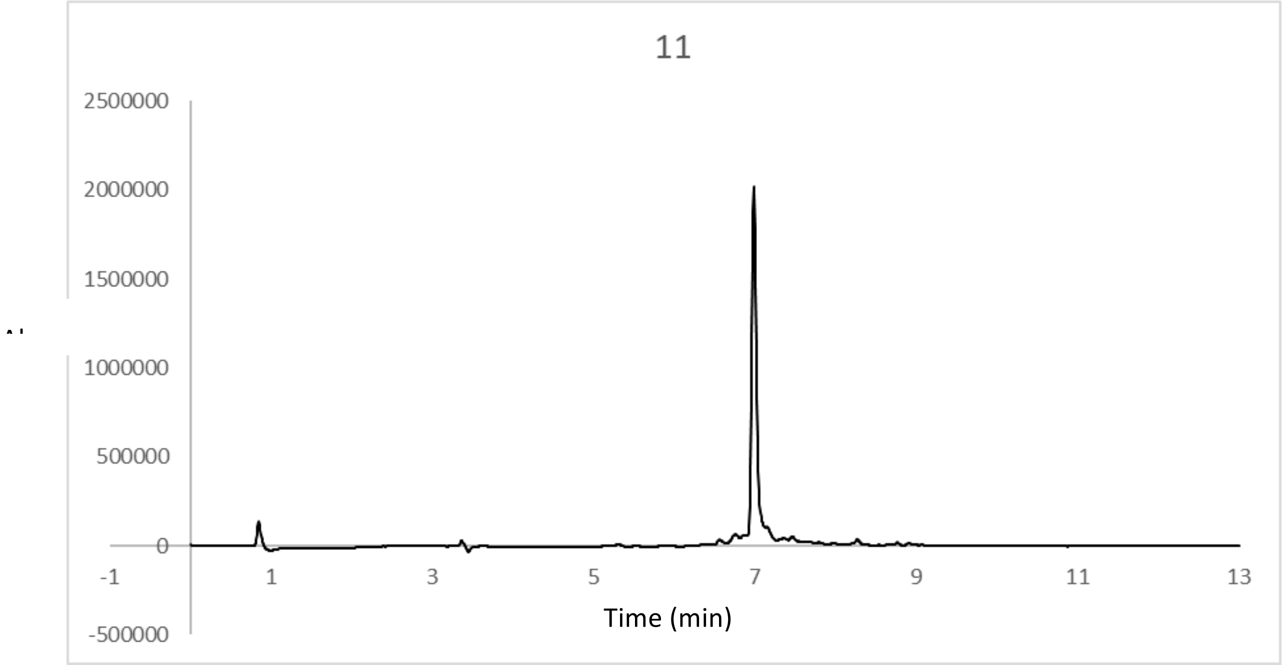

11
2500000
2000000
1500000
1000000
500000
0
-500000
-1
1
3
5
7
9
11
13
Time (min)

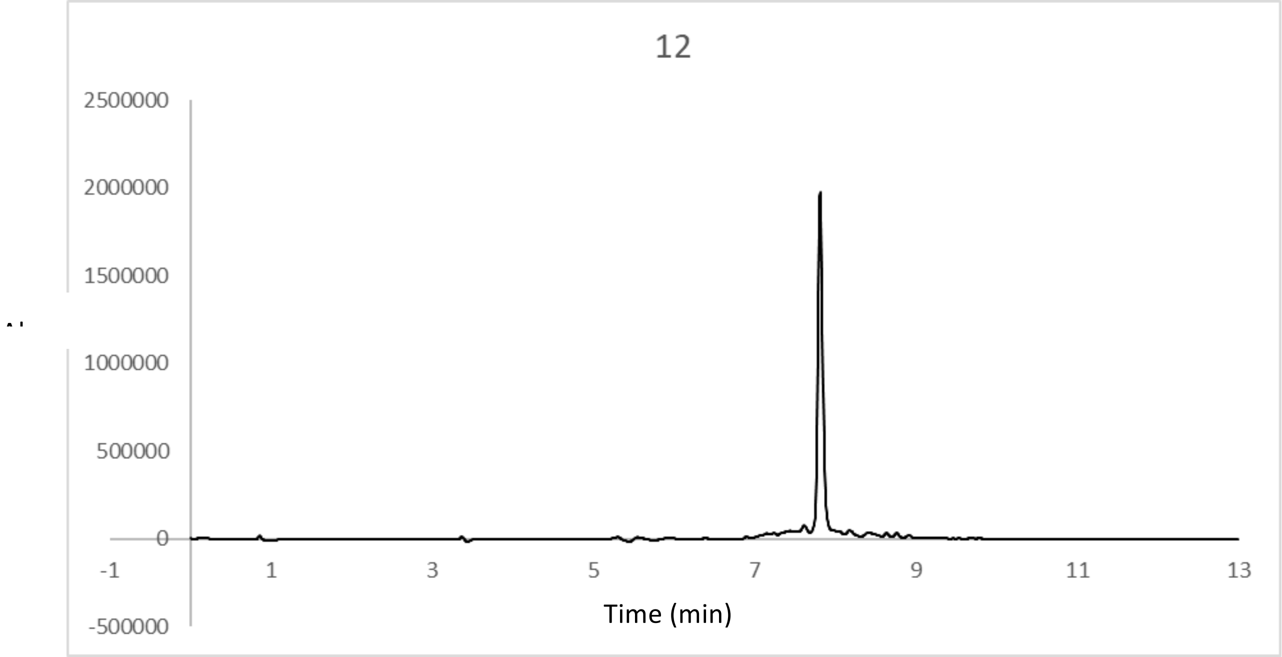

12
2500000
2000000
1500000
1000000
500000
0
-500000
-1
1
3
5
7
9
11
13
Time (min)

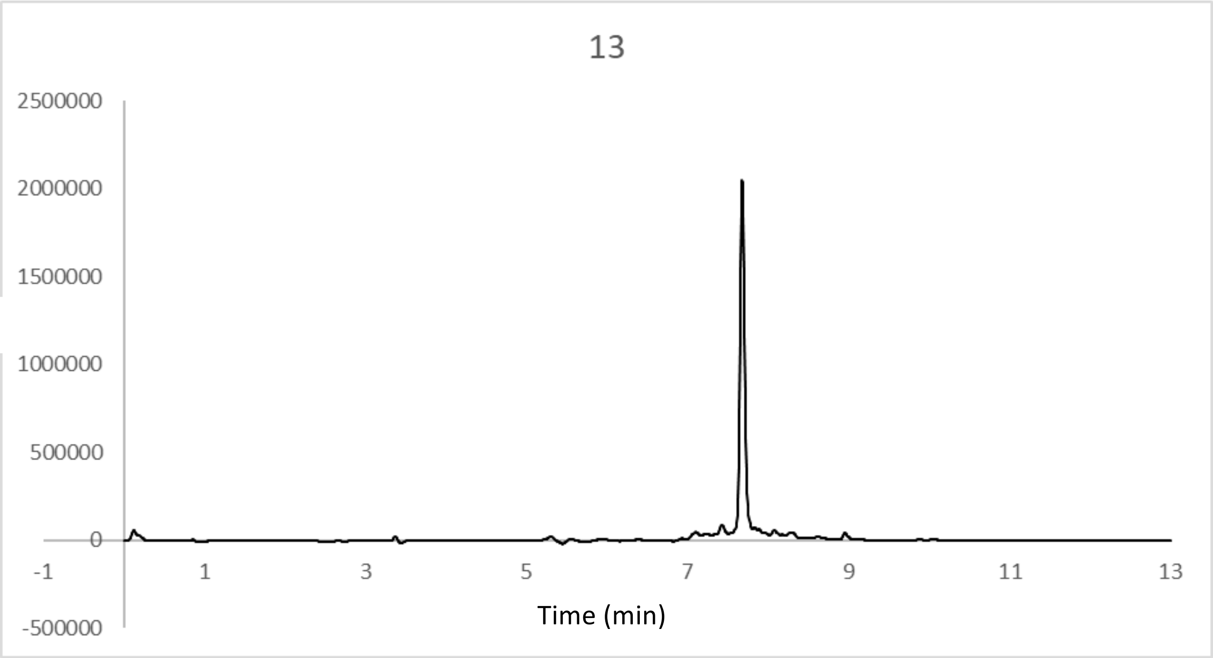
13
2500000
2000000
1500000
1000000
500000
0
-500000
-1
1
3
5
7
9
11
13
Time (min)

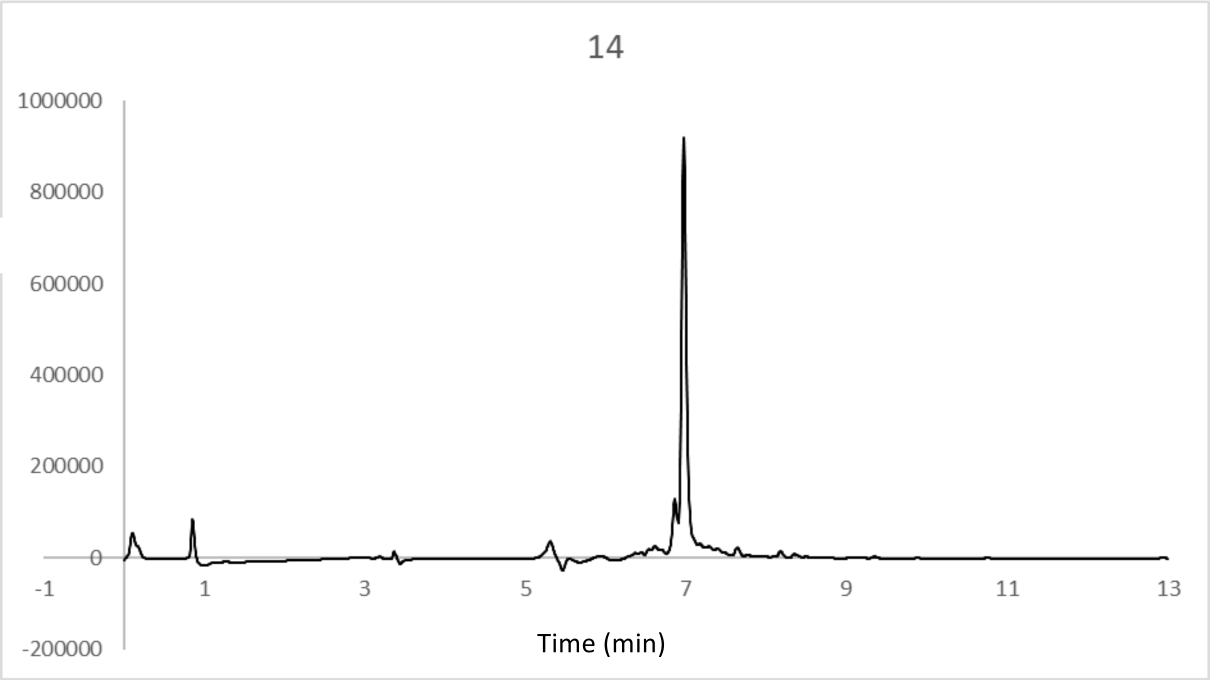
14
1000000
800000
600000
400000
200000
0
-200000
-1
1
3
5
7
9
11
13
Time (min)

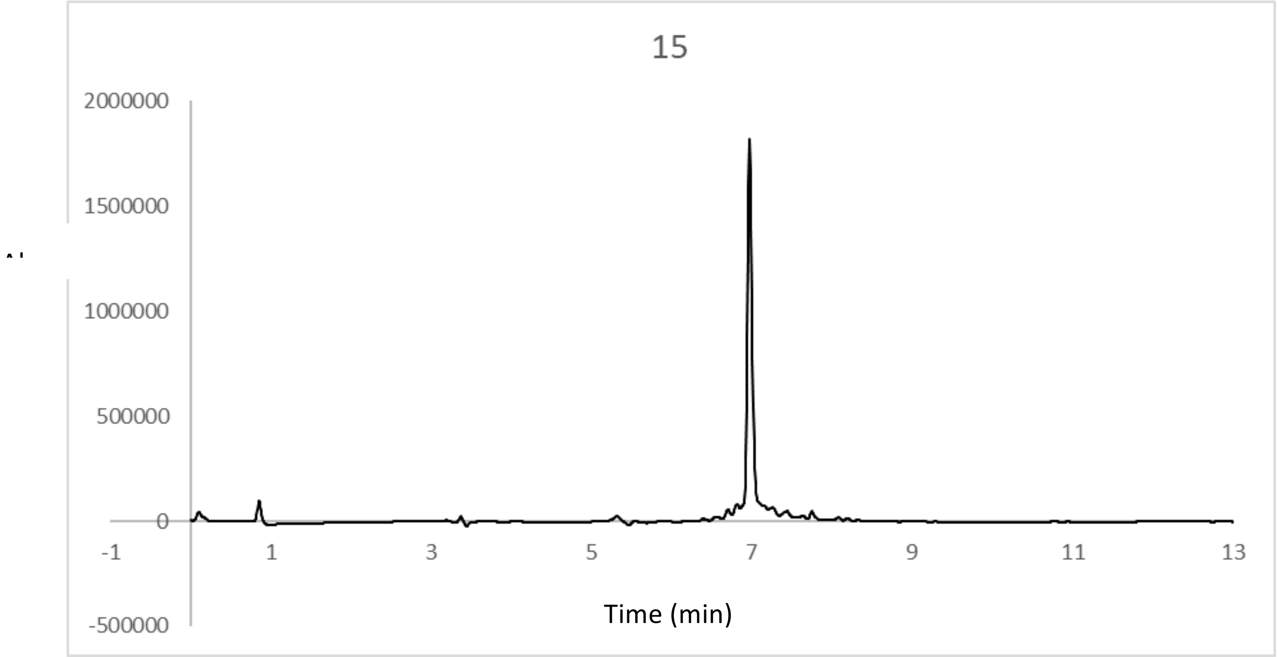
15
2000000
1500000
1000000
500000
0
-500000
-1
1
3
5
7
9
11
13
Time (min)

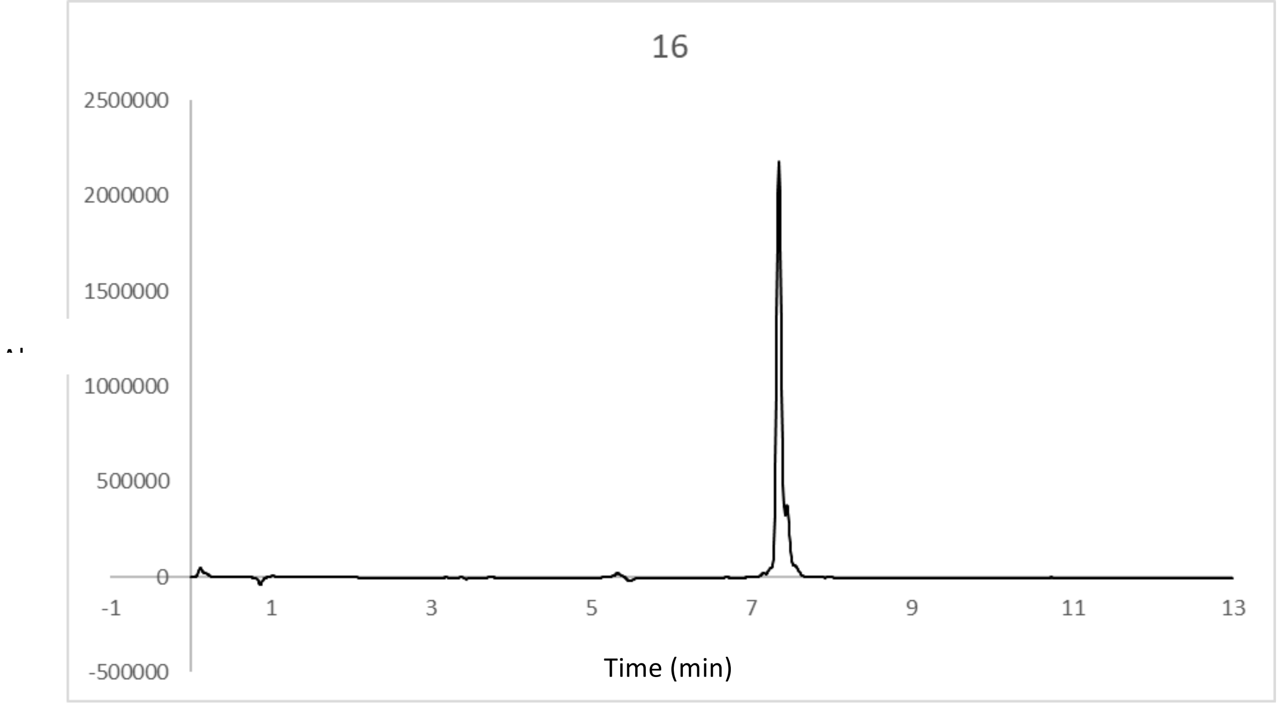
16
2500000
2000000
1500000
1000000
500000
0
-500000
-1
1
3
5
7
9
11
13
Time (min)

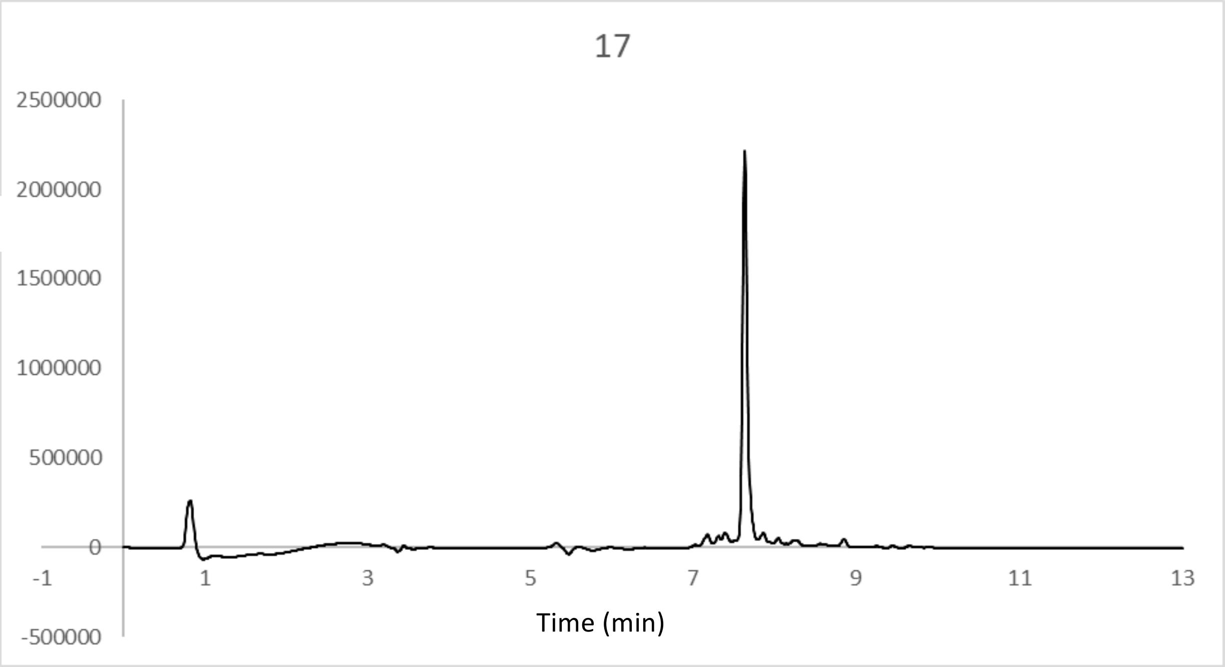
17
2500000
2000000
1500000
1000000
500000
0
-500000
-1
1
3
5
7
9
11
13
Time (min)

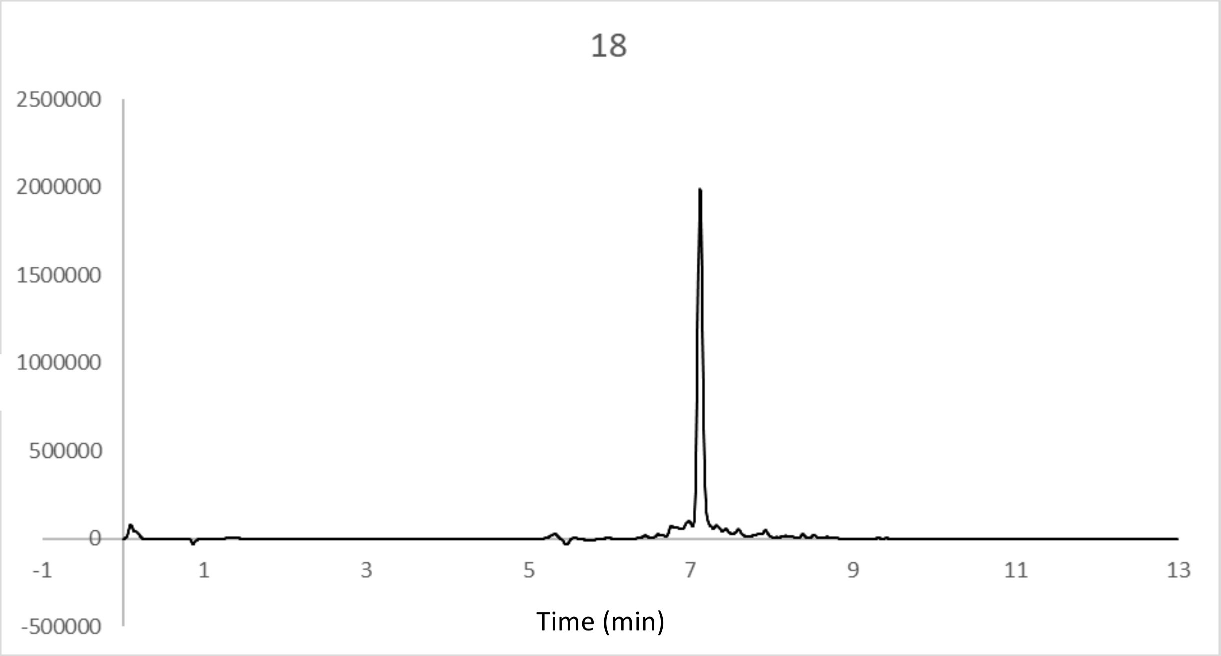
18
2500000
2000000
1500000
1000000
500000
0
-500000
-1
1
3
5
7
9
11
13
Time (min)

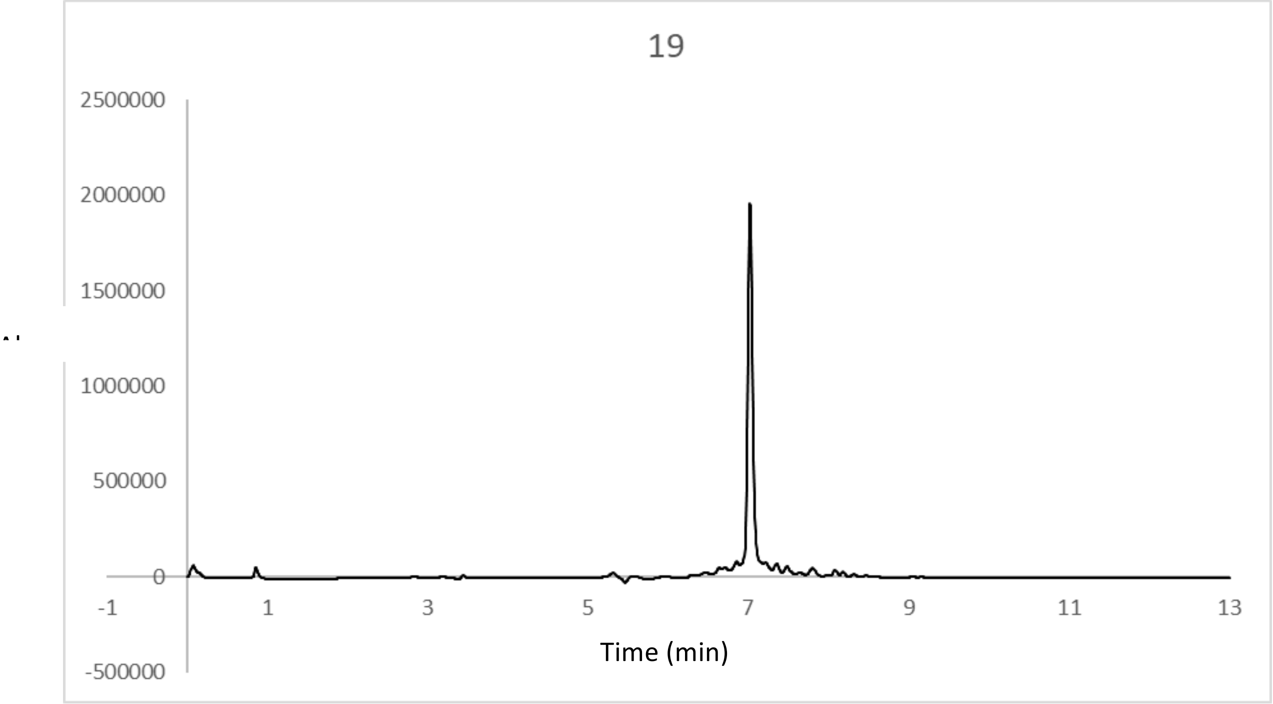
19
2500000
2000000
1500000
1000000
500000
0
-500000
-1
1
3
5
7
9
11
13
Time (min)

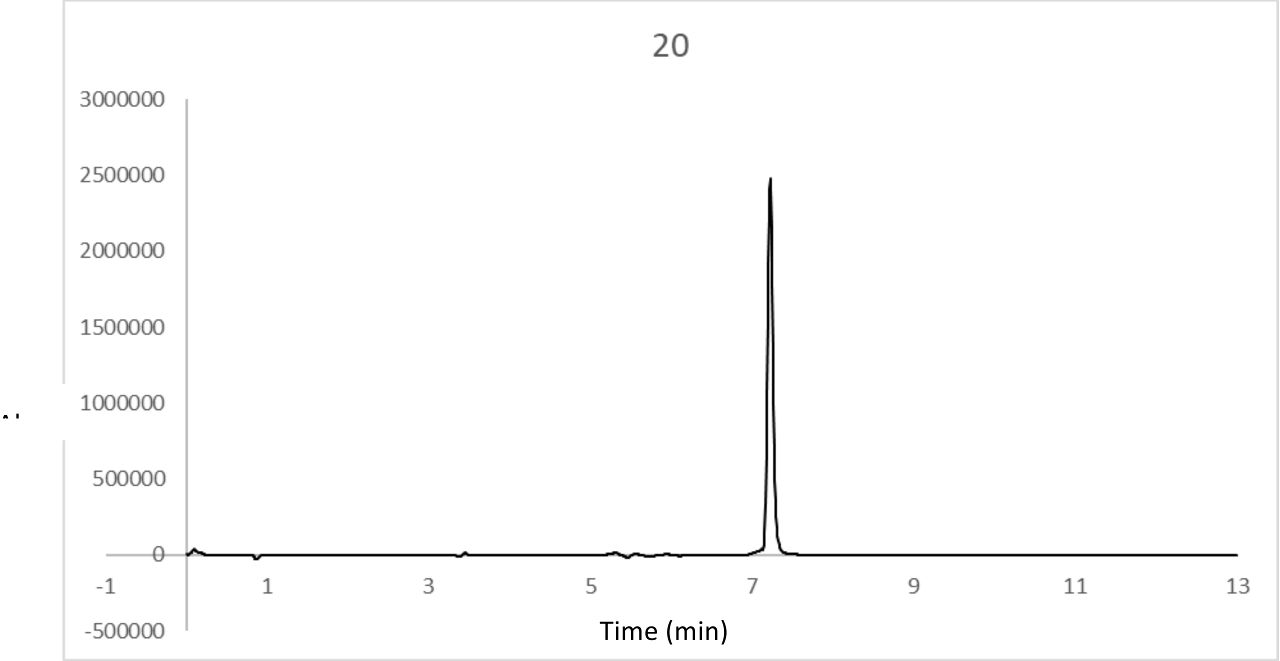
20
3000000
2500000
2000000
1500000
1000000
500000
0
-500000
-1
1
3
5
7
9
11
13
Time (min)

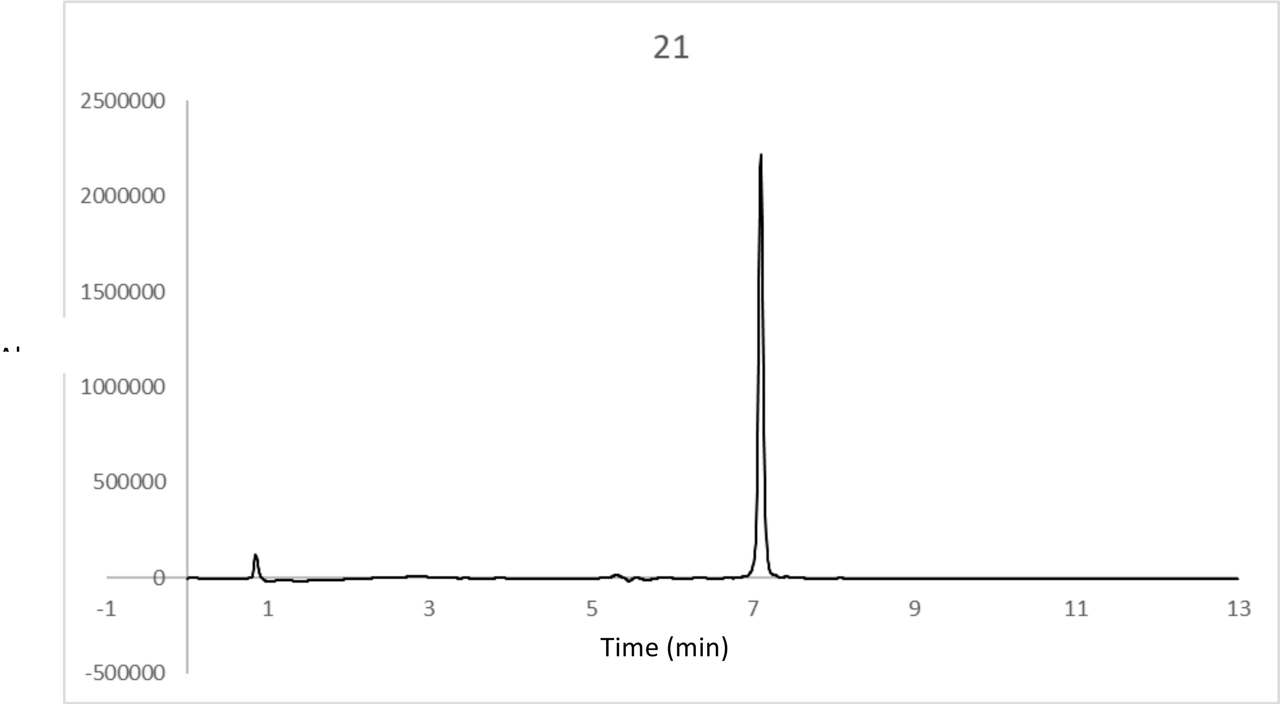

21
2500000
2000000
1500000
1000000
500000
0
-500000
-1
1
3
5
7
9
11
13
Time (min)

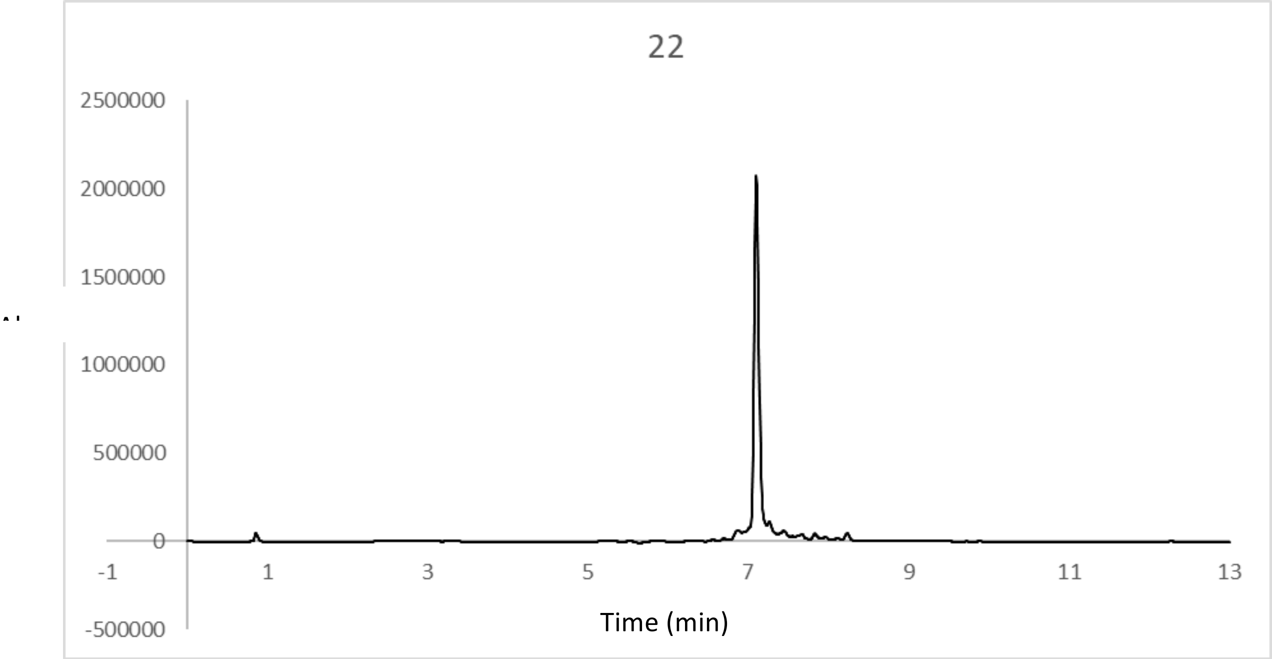

22
2500000
2000000
1500000
1000000
500000
0
-500000
-1
1
3
5
7
9
11
13
Time (min)

Sources of bacterial strains

Utrecht University Medical Center (UMC), Microbiology department, Heidelberglaan 100, 3584 CX Utrecht, The Netherlands

E.coli BW25113

E.coli 552060.1

E.coli mcr-1

Leiden University Medical Center (LUMC), Department of Medical Microbiology, Albinusdreef 2, 2333 ZA Leiden, The Netherlands

A. baumannii ATCC17978

E.coli ATCC25922

K. pneumoniae ATCC13883

P. aeruginosa ATCC27853

Wageningen Bioveterinary Research, Bacteriology and Epidemiology, Houtribweg 39, 8221 RA Lelystad, The Netherlands

E. coli EQASmcr-1/EQAS 2016 412016126

E. coli EQASmcr-2/EQAS 2016 KP37

E. coli EQASmcr-3/EQAS 2017 2013-SQ352

Utrecht University, Molecular Pharmacy, Universiteitsweg 99, 3584 CG Utrecht, the Netherlands

S. aureus ATCC29213

References

1. Odds, F. C. Synergy, antagonism, and what the chequerboard puts between them. *J. Antimicrob. Chemother.* **2003**, *52*, 1–1.

2. Kasetty, G.; Papareddy, P. Structure-activity studies and therapeutic potential of host defense peptides of human thrombin. *Antimicrob. Agents Chemother.* **2011**, *55*, 2880–2890.

3. MacNair, C.R.; Stokes, J.M. Overcoming *mcr-1* mediated colistin resistance with colistin in combination with other antibiotics. *Nat. Commun.* **2018**, *9*, 548.
